# Heterointerface‑Enabled Electrocatalysis for Efficient Energy Conversion

**DOI:** 10.1002/advs.75065

**Published:** 2026-04-09

**Authors:** Liuru Fang, Hsiwen Wu, Hengyue Xu, Jie Zhang

**Affiliations:** ^1^ School of Chemistry Monash University Clayton Victoria Australia; ^2^ ARC Research Hub for Carbon Utilisation and Recycling Monash University Clayton Victoria Australia; ^3^ ARC Centre of Excellence for Green Electrochemical Transformation of Carbon Dioxide Monash University Clayton Victoria Australia; ^4^ Department of Chemistry Tsinghua University Beijing China

**Keywords:** Electrocatalysis, Energy conversion, Heterostructured electrocatalysts

## Abstract

Electrochemical processes hold significant promise for renewable‑energy conversion and decreasing the reliance on fossil fuels. Progress in this field hinges on efficient and durable electrocatalysts, which has spurred immense research efforts in catalyst design and engineering. Heterointerface has been shown to be an effective strategy in enhancing the performance of electrocatalysts in important electrochemical reactions involving small molecules. This review summarizes how interfacial structures influence charge‑transfer dynamics, active‑site identity and its density, catalytic activity, and long‑term stability. Interface‑induced effects are first introduced, followed by a survey of common synthetic strategies for heterostructured electrocatalysts. The role of heterointerface in electrocatalysis is discussed using the representative electrochemical conversion of small molecules. We highlight recent progress across energy‑conversion reactions, focusing on interface‑enabled functionalities, and systematically evaluate strategies and performance across diverse electrocatalytic contexts. Finally, we discuss future challenges and opportunities—including innovations in synthesis, advanced characterization, and theory and computation—to guide rational interface design. Overall, this review provides actionable insights for heterostructured electrocatalysts in energy conversion and supports progress toward carbon neutrality.

## Graphical abstract

Functionally distinct A/B materials can be integrated at the nanoscale to create customized heterointerfaces with tunable band alignment and charge redistribution, providing fast electron and ion transport channels as well as cooperative dual active sites. This design markedly enhances the activity and selectivity of oxygen reduction and evolution, hydrogen evolution and oxidation, nitrogen reduction, carbon dioxide reduction, and C─N coupling reactions.

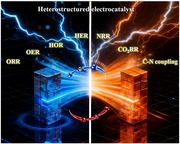

## 1 Introduction

**FIGURE 1:**
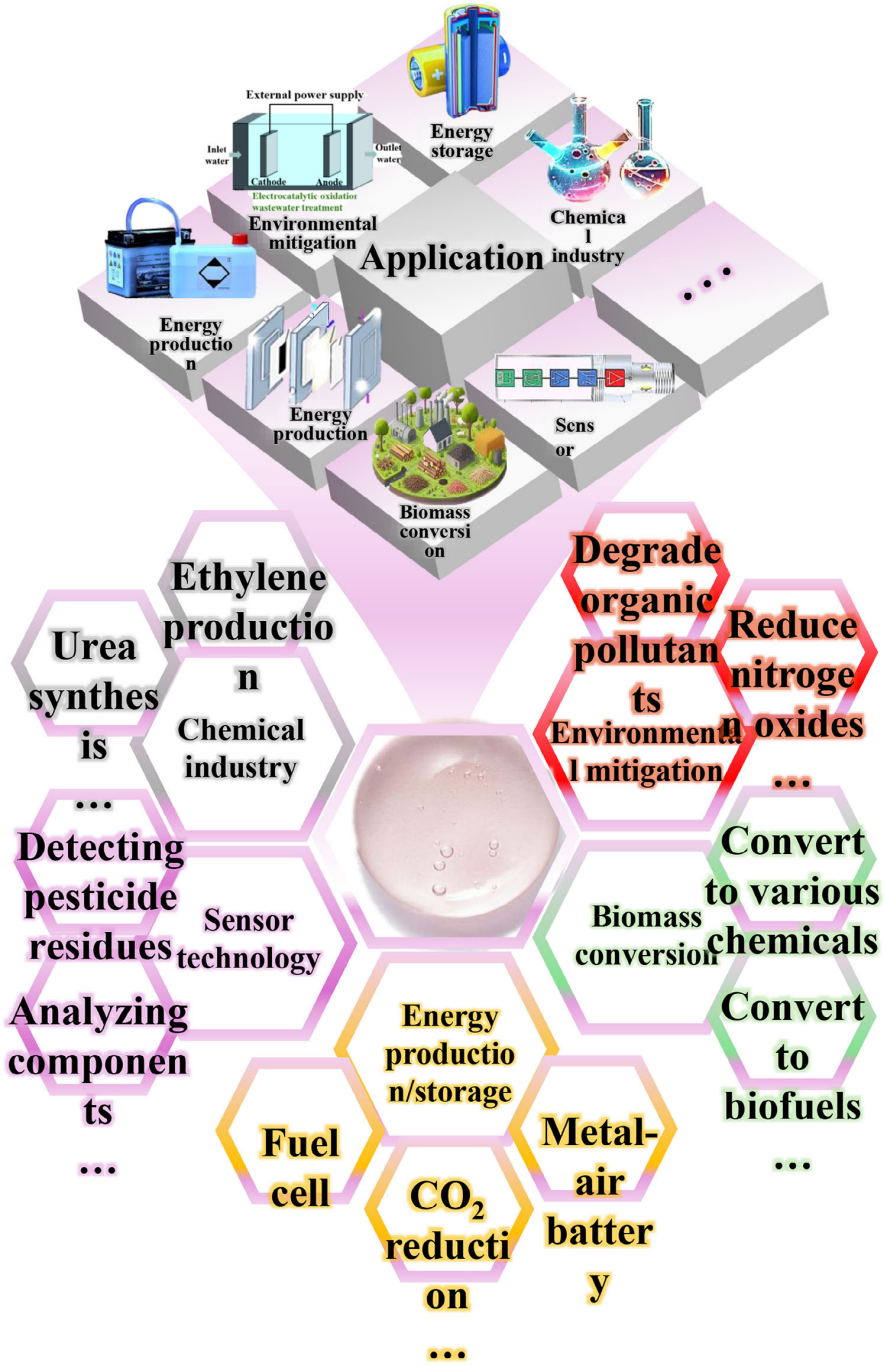
Key applications of electrocatalysis.

Energy and environmental challenges have raised global concerns, prompting scientists to seek cleaner, more sustainable energy solutions [1, 2, 3]. Electrocatalysis is a pivotal route to efficient energy conversion and storage, underpinning the widespread deployment of renewable energy sources [4, 5]. Figure 1 illustrates applications of electrocatalysis across multiple fields, in which energy conversion and storage play central roles in the sustainability of these processes. Energy storage technologies convert transient energy sources, such as solar and wind energy, into storable forms, such as chemical bonds in battery materials or fuels produced electrolysis. Energy conversion technologies transform stored chemical energy into electricity to meet immediate demand (e.g., fuel cells and batteries) [6, 7]. Electrocatalysts are central to these processes, of which the key performance metrics, such as energy efficiency, product selectivity, and durability, depend critically on the effectiveness and stability of the electrocatalysts employed. Over the past decades, significant efforts have focused on developing advanced electrocatalysts to deliver more efficient and durable operation. Currently, precious metals—notably platinum, iridium, and ruthenium‑based materials—are among the most effective electrocatalysts for the hydrogen evolution reaction (HER) and oxygen evolution reaction (OER). However, their high cost, limited natural abundance, and potential environmental impacts associated with mining and extraction hinder their large‑scale application. In addition, many electrochemical reactions are limited by sluggish kinetics, leading to increased overpotentials and reduced stability. Furthermore, competing reactions and materials instability can confine operation to narrow redox windows, undermining the overall efficiency and selectivity [8, 9, 10, 11]. Therefore, designing low‑cost electrocatalysts to reduce overpotential, promote electrode reactions, and enhance long‑term stability is the goal of advancing electrochemical technologies. Optimization of structure, morphology, and composition can increase active‑site density and intrinsic activity, thereby enhancing electrocatalytic activity, stability, and selectivity [12, 13]. Strategies such as surface modification, heterostructure construction, doping, and alloying have been widely used to improve electrocatalyst performance [14, 15, 16, 17, 18, 19].

Heterostructure construction constructs well‑defined interfaces (i.e., atomistic arrangement within the contact region of distinct phases) between different materials. Such interfaces enable precise control of the state and transport of charge‑carriers and have been widely exploited in electronics, spintronics, and optoelectronics to design advanced devices [20, 21, 22, 23]. In a typical interfacial coupling, rebalancing of the Fermi levels and work functions (Δφ) of the two materials occurs at the interface. Upon Fermi‑level equilibration, a band alignment is established; depending on the difference in work function, the junction behaves as a Schottky (rectifying) or Ohmic contact [24, 25]. By tailoring interfacial electronic structure through charge‑carrier redistribution, electrocatalytic kinetics and selectivity can be significantly improved [26, 27, 28, 29]. Across key electrocatalytic processes—oxygen reduction/evolution (ORR [30]/OER [31, 32]), hydrogen evolution/oxidation (HER [33]/HOR [34]), nitrogen reduction (NRR [35]), and CO_2_ reduction (CO_2_RR [36]).

The central obstacles are the design of efficient, cost‐effective interfacial architectures, the catalyst stability under realistic operating conditions, and the translation of laboratory advances to commercial deployment. In addition, for complex electrocatalytic mechanisms, clarifying how the modified catalyst surface directs reaction pathways and controls selectivity remains a central research focus. Therefore, a comprehensive summary of the current state and outlook of the field is warranted. Existing landmark reviews have covered diverse aspects of electrocatalysis—ranging from efficient hydrogen and oxygen production in electrolyzers and mechanistic analyses [37], to facet‐controlled noble‑metal nanocrystals in catalysis/electrocatalysis [38], to in situ/operando microscopy and spectroscopy correlating structure and performance [39], interfacial interactions among catalyst, electrolyte, and electrode [40], and interfacial‐structure‐reaction‐mechanism relationships in CO_2_RR and ORR [41, 42]. However, few reviews have systematically detailed the application of heterostructured electrocatalysts across ORR, OER, HOR, HER, CO_2_RR, and NRR. Accordingly, this review (i) analyzes how interfacial structures influence charge‑transfer dynamics, active‑site density and identity, catalytic activity, and stability; (ii) surveys common synthetic methods for heterostructured electrocatalysts; and (iii) summarizes recent progress across ORR, OER, HOR, HER, CO_2_RR, and NRR, highlighting composition‑dependent performance and interface‑enabled functionalities. Finally, we outline current limitations in heterostructured electrocatalysts and the prospective research directions in this area. In short, while heterostructures show great promise for electrocatalysis, challenges remain in commercial translation, deeper understanding of interfacial effects, and integration with other functional materials. Future research should clarify working mechanisms, develop scalable and green synthetic strategies, and deliver more efficient, durable heterostructured electrocatalysts for energy conversion, environmental protection, and sustainable development. Through this review, we aim to provide valuable references and insights to advance efficient, stable electrocatalysts.

## 2 The Role of Heterointerface in Electrocatalysis

**FIGURE 2:**
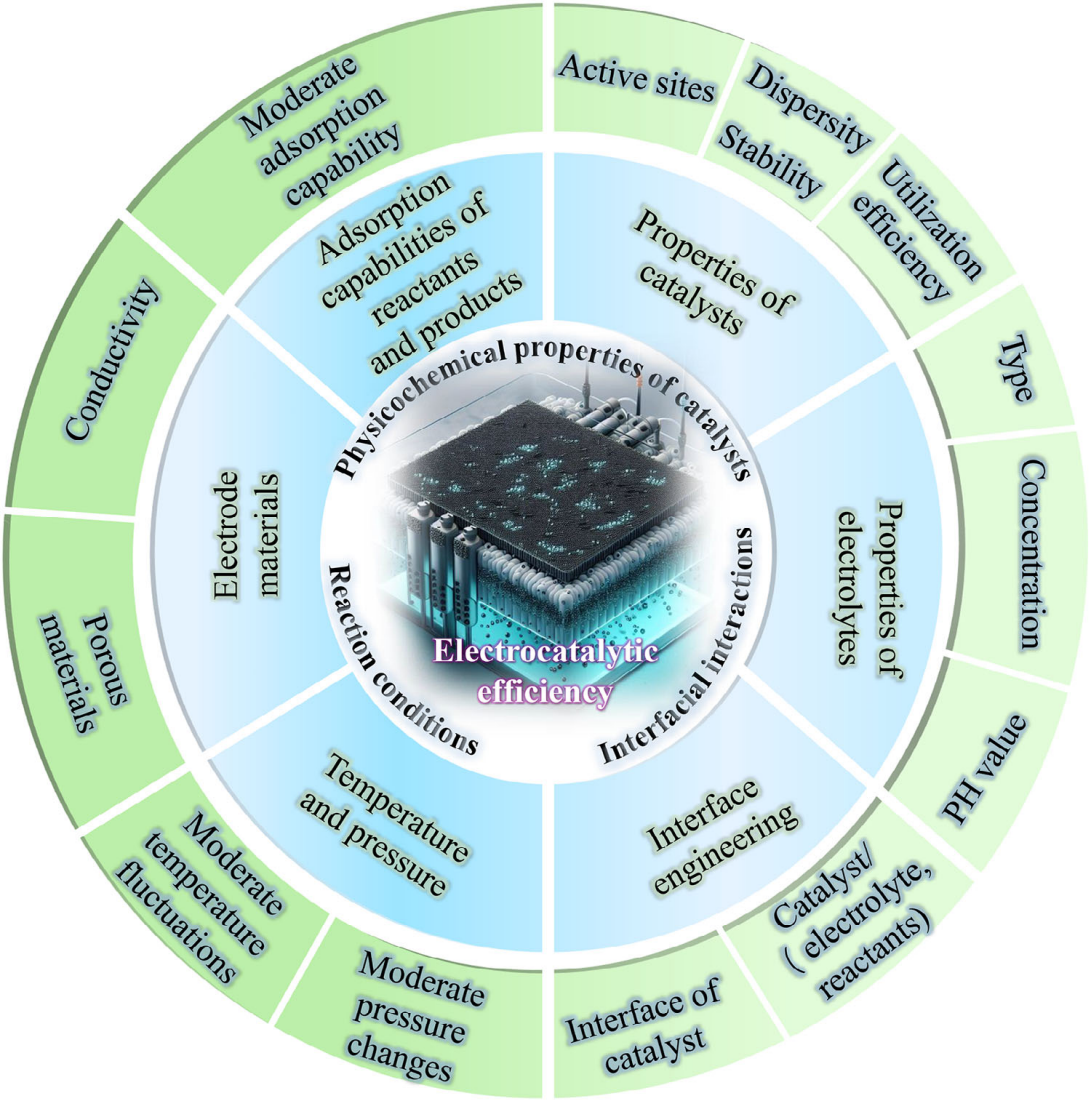
A schematic illustration of some common factors affecting the efficiency of electrocatalysis.

Electrocatalysis comprises a series of reactions occurring at the electrochemical interface, where electron transfer mediates the transformation of chemical species [43, 44]. The rate and efficiency of these reactions are governed by multiple factors, including the catalyst's electronic structure, the density and nature of active sites, electrolyte composition (e.g., pH and ionic strength, where relevant), and operating conditions such as applied potential, temperature, and pressure, etc. (Figure 2). By modulating these factors, the performance of electrocatalytic reactions can be optimized. An electrocatalyst functions by lowering the activation barrier, thereby accelerating reaction rates and improving overall energy‑conversion efficiency [45, 46, 47]. The electronic structure and surface properties (e.g., hydrophobicity, geometric and steric effects) of the electrocatalyst are especially critical [48], as they dictate the adsorption, activation, and desorption of reactant and reaction intermediate molecules. Mechanistically, electrocatalysis typically proceeds through several steps: (i) charge transfer from the external circuit through the electrode/support to the catalytic active site—often as proton‑coupled electron transfer (PCET) and governed by the interfacial resistance (iR) and the electronic conductivity across the heterojunction; (ii) adsorption and desorption—occupation and regeneration of active sites; (iii) surface reactions (may include electrochemical and chemical reaction) in which the adsorbates are converted to products through bond‑forming/breaking of the reaction intermediates, completing the catalytic cycle [49, 50, 51, 52]. A clear understanding and control of these processes enables the rational design of efficient and durable electrocatalysts for fuel cells, water electrolysis, and other electrochemical energy‑conversion devices.

To boost electrocatalytic efficiency, diverse electronic‑structure engineering strategies have been developed. For example, doping and alloying tune the energy‑level structure and Fermi level of electrocatalysts, creating new active sites or optimizing existing ones to improve adsorption/desorption and electron transfer [53, 54, 55, 56]. Building on the electronic‑structure tuning discussed above, heterojunction engineering provides a complementary lever at the catalyst surface. Constructing interfaces between dissimilar materials establishes controllable band alignment and low‑resistance contact in the near‑surface region, thereby increasing the electronic conductivity across the heterojunction and shifting the adsorption free energies of key intermediates—effects that correlate with higher activity [57]. In parallel, architecting electronically conductive supports with tailored morphologies (e.g., porous frameworks) increases the electrochemically accessible surface area (ECSA) and the density of accessible active sites, improving the apparent rate without conflating it with intrinsic kinetics [58]. Practically, electrochemical deposition—through control of the potential waveform/pulse scheme, precursor concentration, and temperature—enables precise tuning of junction thickness, coverage, and composition, linking synthesis parameters to measurable junction descriptors (charge‑transfer resistance(*R*_ct_), ECSA, active‑site density) and thus to performance [59, 60, 61]. Surface modification with selected functional groups or ligands further tailors the local electronic environment and, in turn, the reaction pathway and selectivity [62, 63, 64]. In addition, strong metal–support interactions can redistribute charge and optimize the catalyst's electronic structure, thereby improving activity [65, 66, 67, 68, 69]. This review focuses on electronic tuning of electrocatalyst through heterostructure engineering, and covers the working principles and recent monumental progress in this area. The electronic structure and surface properties of the catalyst jointly determine electrocatalytic activity by governing surface–adsorbate interactions (adsorption, activation, and desorption of reactants and intermediates) and the associated reaction energy landscape [70]. Active‑site density per unit area, together with the ECSA, determines the total site number, while their geometric arrangement and electronic character govern affinity toward specific reactants [71]. From an electronic‑structure perspective, the band structure determines the density of states (DOS) and carrier mobility [72]. Appropriate band alignment within heterojunctions enhances electronic conductivity across the interface, facilitating charge transport that supports overall redox processes [73]. Shifting the Fermi level modulates surface electron density [74], thereby tuning adsorption and activation of reactants. Defect sites such as vacancies, dislocations, and grain boundaries (GBs) may also act as highly reactive centers by providing local environments distinct from the perfect lattice. Surface features (facets, edges, and corners) profoundly influence adsorption modes and reaction pathways; for example, some facets exhibit higher adsorption energies for specific reactants and thus better activate them. Functional‑group modification alters the surface electronic environment and so steers reaction pathways and selectivity [75, 76]. Importantly, electronic structure and surface geometry act synergistically: tuning one often changes the other's effect on activity.

In materials relevant to energy conversion and chemical synthesis, unique electronic and geometric attributes determine performance. Grain boundaries are interfaces between crystallites, and these often possess higher energy owing to lattice mismatch [77]. They increase effective surface area (exposing more sites) and, due to locally irregular atomic arrangements, stabilize key intermediates to lower activation barriers; dislocations and steps at GBs frequently serve as adsorption sites that promote reactivity. In heterojunction electrocatalysts, differences in electronic properties between phases create built‑in electric fields that aid electron–hole separation near the catalyst surface, which, when appropriately designed, enables improved electronic conductivity and accelerated charge‐transfer kinetics. In the electrocatalyst interface, electronic conductivity is governed by band alignment and the interfacial DOS. Electroneutrality during turnover is preserved by ionic flux in the electrolyte/ionomer (e.g., proton hopping or migration of other mobile ions) that balances the electronic current at the interface and closes the circuit under steady‑state operation [78, 79, 80]. Accordingly, precise control over interfacial composition, structure, and electronic properties can optimize charge‑transfer pathways and improve catalytic efficiency. For instance, introducing dopants or designing special interfacial architectures allows one to tune the strength and polarity of the built‑in field and thus the efficiency of electron–hole separation. Interfacial functional‑group modification together with metal and non‑metal heteroatom doping improves lattice matching and chemical compatibility, tunes surface electronic structure, and thus enhances adsorbate adsorption/desorption and performance. These principles have been demonstrated in numerous studies, where heterostructured materials show optimized adsorption energetics and higher interfacial electronic conductivity—via favorable band alignment, interfacial DOS, and intimate contact (lower R_ct_)—together with improved stability over single‐component counterparts [81]. Therefore, in what follows, we summarize how heterostructures enhance these catalytic attributes to guide future research.

### 2.1 Promoting Electron Transfer

Rapid electron transfer is essential for enhancing reaction kinetics and minimizing energy loss during the electrocatalytic process. Catalysts with high electronic conductivity primarily lower ohmic (iR) losses across the electrode–electrolyte–catalyst network, while activation losses (intrinsic kinetics) and concentration losses (mass transport) are governed by different factors. Moreover, one‑ and two‑dimensional nanostructures (e.g., nanowires, nanotubes, or nanosheets) serve as directed conduits for electron transfer, providing higher interfacial electronic conductivity. At the same time, new interfacial electronic states (e.g., interfacial or surface states) mediate charge transport and increase carrier mobility. In addition, quantum‑mechanical electron tunneling and strong electronic coupling at the interface increase interfacial electronic conductivity (lower R_ct_), enabling rapid charge delivery under operating conditions. External driving forces—electric fields, light, or targeted chemical modification—tune interfacial electronic states; lattice strain reduces electron–phonon scattering and increases carrier mobility; and control of carrier concentration and its spatial distribution sets the Fermi level and screening length, adjusting interfacial band bending and the width of the space‑charge region.

### 2.2 Regulating Electronic Structure

Regulation of the electronic structure plays a central role in heterostructure catalysts, and below introduces some of the strategies that utilize electronic regulation. Surface modification alters the surface electronic environment by introducing functional groups or ligands to steer the catalytic reaction pathway. Band‑structure engineering modulates the bandgap, affecting electron excitation and transfer. Band bending at the heterojunction, arising from differences in electron affinity between components, creates a built‑in electric field that promotes charge‑carrier (electron–hole) separation. When the junction is located within the near‑surface screening/depletion length of the catalytic layer (0.1–0.3 nm for metals, 1–5 nm for conductive oxides/carbides/nitrides, 5–20 nm for moderately doped semiconductors), it directly modulates the surface electronic structure and thus the adsorption/activation energetics. The near‑surface DOS is tuned by composition and defect chemistry, and by the thickness/domain size of the surface layer (e.g., shell thickness, grain/domain size), which together set the population and energies of active sites. Interfacial charge transfer and dipole formation modify the electronic environment, shaping catalytic reactions. Interface‑induced strain regulation uses stress caused by lattice mismatch and, when operation is non‑ambient or during thermal processing, by differences in thermal expansion coefficients to adjust the electronic structure.

### 2.3 Enhancing Interfacial Effects

The formation of heterojunction involves intertwined physical and chemical mechanisms—including lattice mismatch, differences in electron affinity, chemical‑bond formation, and stress‑induced effects [82]. Collectively, these factors drive interfacial charge redistribution and create new active sites. The promotional effect of heterojunction on electrocatalytic performance is mainly reflected in: (i) enhanced interfacial electronic conductivity, (ii) optimized adsorption of reactants and intermediates, (iii) improved catalyst stability, and (iv) regulation of reaction pathways. To achieve precise control of interface effects, the following strategies can be adopted: (a) adjusting the interfacial chemical composition, (b) designing specific interface architectures, (c) reinforcing interfacial stability, and (d) tuning interfacial electronic states—specifically the working oxidation state and coordination environment.

### 2.4 Providing Abundant Active Sites

Abundant active sites increase effective contact between the electrolyte/reactants and the catalyst, thereby accelerating the electrocatalytic process. Through rational interfacial engineering of heterojunctional catalysts, the domain size and topology can be controlled and integrated with high‑surface‑area supports, thus exposing more active sites. However, nano‐materials are prone to agglomerate, especially during electrocatalysis, decreasing the ECSA and the number of accessible active sites. This aggregation lowers the apparent current density at a given potential and increases charge‑transfer/series and mass‑transport losses, thereby requiring higher overpotentials to reach a target rate. By contrast, strong interfacial binding in heterostructured catalysts, either between two catalysts or between catalyst and support, suppresses aggregation and delamination, thus maintaining a high density of exposed active sites. These considerations align with the factors summarized in Figure 2.

Although heterojunction catalysts show great promise, their design and application still face many challenges [83]. The central challenge is achieving and maintaining high site utilization under realistic operation. Interfacial stability can degrade during prolonged electrolysis via debonding, ripening, or dissolution; accordingly, retention of ECSA and R_ct_ should be reported over extended tests together with post‑mortem morphology to diagnose failure modes. Going forward, synthesis that links junction thickness/coverage and defect chemistry to measurable descriptors (ΔG of key intermediates, the exchange current density (*j*_0_), Tafel slope, R_ct_), combined with operando probes of working oxidation/coordination states, will enable rational interface design and durability at device‑relevant current densities [84].

## 3 Methods for Constructing Interface Structures

Heterostructures are two or more compositionally and/or crystallographically distinct domains integrated within a single construct, thereby forming an intimate heterointerface [85]—the atomically proximate contact region between domains that enables electronic and/or ionic coupling. We use the term heterojunction more specifically when the interface exhibits a discernible junction effect—such as band alignment and built‐in electric fields that lead to Schottky‐ or Ohmic‐type behavior, or the emergence of interfacial electronic states—with measurable consequences for charge transport and adsorption energetics. Accordingly, a simple physical mixture of two phases is not necessarily a heterostructure in the mechanistic sense unless the domains are electrically connected and the interfacial contact is sufficiently intimate to induce charge redistribution and cooperative adsorption/activation under catalytic conditions.

Reliable verification of heterostructure formation should be based on converging evidence that establishes nanoscale domain adjacency, correct phase assignment, and interfacial chemical or electronic coupling. Direct structural confirmation is most convincingly obtained by high‐resolution transmission electron microscopy (HRTEM) and aberration‐corrected high‐angle annular dark‐field scanning transmission electron microscopy (HAADF‐STEM), ideally combined with energy‐dispersive X‐ray spectroscopy (EDS) or electron energy loss spectroscopy (EELS) mapping or line profiling to resolve compositional gradients across the interface. Phase identification is commonly supported by X‐ray diffraction (XRD) and selected‐area electron diffraction (SAED), together with fast Fourier transform (FFT) analysis of lattice images. However, these ensemble approaches alone are usually insufficient to quantify interfacial density, accessibility, or electrical connectivity. Evidence for interfacial chemical and electronic coupling can be derived from X‐ray photoelectron spectroscopy (XPS) core‐level shifts, X‐ray absorption spectroscopy (XAS)—including extended X‐ray absorption fine structure (EXAFS) to probe coordination and oxidation‐state evolution—and work‐function or band‐edge measurements using ultraviolet photoelectron spectroscopy (UPS), Kelvin probe force microscopy (KPFM), or Mott–Schottky analysis. Electrochemical diagnostics, particularly electrochemical impedance spectroscopy (EIS) to extract the charge‐transfer resistance (R_ct_) together with kinetic metrics such as Tafel slopes and exchange current density, help connect a validated interface to catalytic consequences through rigorous comparisons against appropriate single‐phase controls. Where feasible, operando/in situ characterization (for example, operando XAS, Raman spectroscopy, and Fourier‐transform infrared spectroscopy (FTIR)) is strongly recommended, because many electrocatalysts undergo bias‐induced reconstruction and the as‐synthesized interface may not represent the true working interface. Finally, density functional theory (DFT) calculations provide an essential complement by quantifying interfacial charge redistribution, electronic‐structure changes, and adsorption free energies of key intermediates, thereby strengthening mechanistic interpretation and helping translate interfacial descriptors into predictive design rules [86]. The methods for preparing heterostructured electrocatalysts are diverse; in this section, we focus on epitaxial growth, heterostructure coatings/arrays, and solvothermal synthesis. These technologies are not only powerful tools for constructing interfaces but also important drivers for advancing the science of electrocatalysis. Through a detailed overview, we show how interfacial engineering optimizes electrocatalytic behavior and opens new avenues to address energy and environmental challenges.

### 3.1 Epitaxy Growth

**FIGURE 3:**
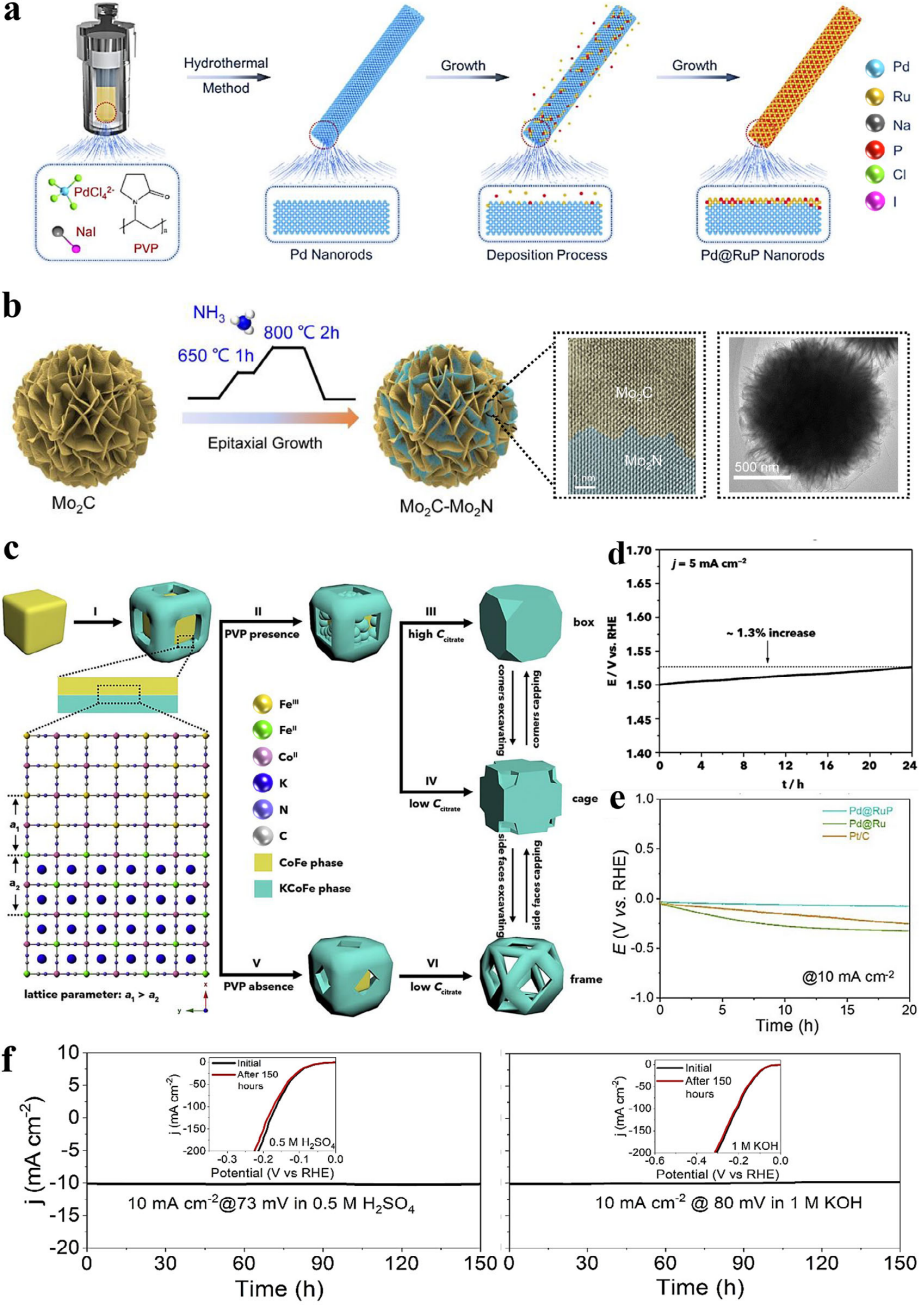
(a) Schematic illustration of the synthesis process for Pd@RuP nanorods (NRs) [85]. Copyright 2023, Elsevier. (b) Schematic representation and microscopy images of the gradient heating epitaxy growth method used to fabricate the Mo_2_C‐Mo_2_N heterostructure. The images include Scanning Electron Microscopy (SEM) and Atomic Resolution Corrected Transmission Electron Microscopy (ACTEM) [89]. Copyright 2023, John Wiley and Sons. (c) Diagram showing the auto‐catalytic crystal growth approach for creating diverse Co–Fe PBA structures, ranging from cages to frameworks and boxes [90]. (d) The electrochemical stability of the Co–Fe–O framework catalyst was assessed through chronopotentiometry at a current density of 5 mA cm^−2^ [90]. (e) Chronopotentiometry curve for the Co–Fe–O framework catalyst measured at a current density of 10 mA cm^−2^ [90]. Copyright 2018, Elsevier. (f) The durability of the Mo_2_C–Mo_2_N heterostructures was evaluated using extended chronoamperometry in both 0.5 M H_2_SO_4_ and 1 M KOH solutions [89].

Epitaxial growth—deposition of a film that maintains a defined crystallographic relationship with a crystalline substrate—has become a key technique in electrocatalytic heterostructure constructing. By precisely controlling deposition conditions, epitaxial growth can produce films with specific orientations on the substrate, enabling atomic‑level control of interface structures. It favors crystallographic registry when the lattice mismatch is small; when the mismatch is larger, strain relaxation accommodates the difference, rather than guaranteeing perfect lattice matching in all cases [87, 88]. Substrate selection is therefore crucial, as it directly affects film quality and interfacial characteristics. Metal substrates are commonly used to facilitate the epitaxial growth of metal catalysts. For instance, a simple hydrothermal route was used to prepare Pd nanorods (NRs) [85], which then served as cores for the epitaxial deposition of an ultrathin RuP alloy shell, yielding Pd@RuP NRs (Figure 3a). Compared with commercial Pt/C electrocatalysts, Pd@RuP NRs deliver a lower overpotential (18 mV at 10 mA cm^−2^) and higher stability for alkaline HER (Figure 3e). Furthermore, a homemade Pd@RuP//RuO_2_ electrolyzer attains 10 mA cm^−2^ at only 1.42 V, highlighting strong potential for practical water electrolysis. This work thus offers an effective approach to designing Ru‑based electrocatalysts via epitaxial growth.

**FIGURE 4:**
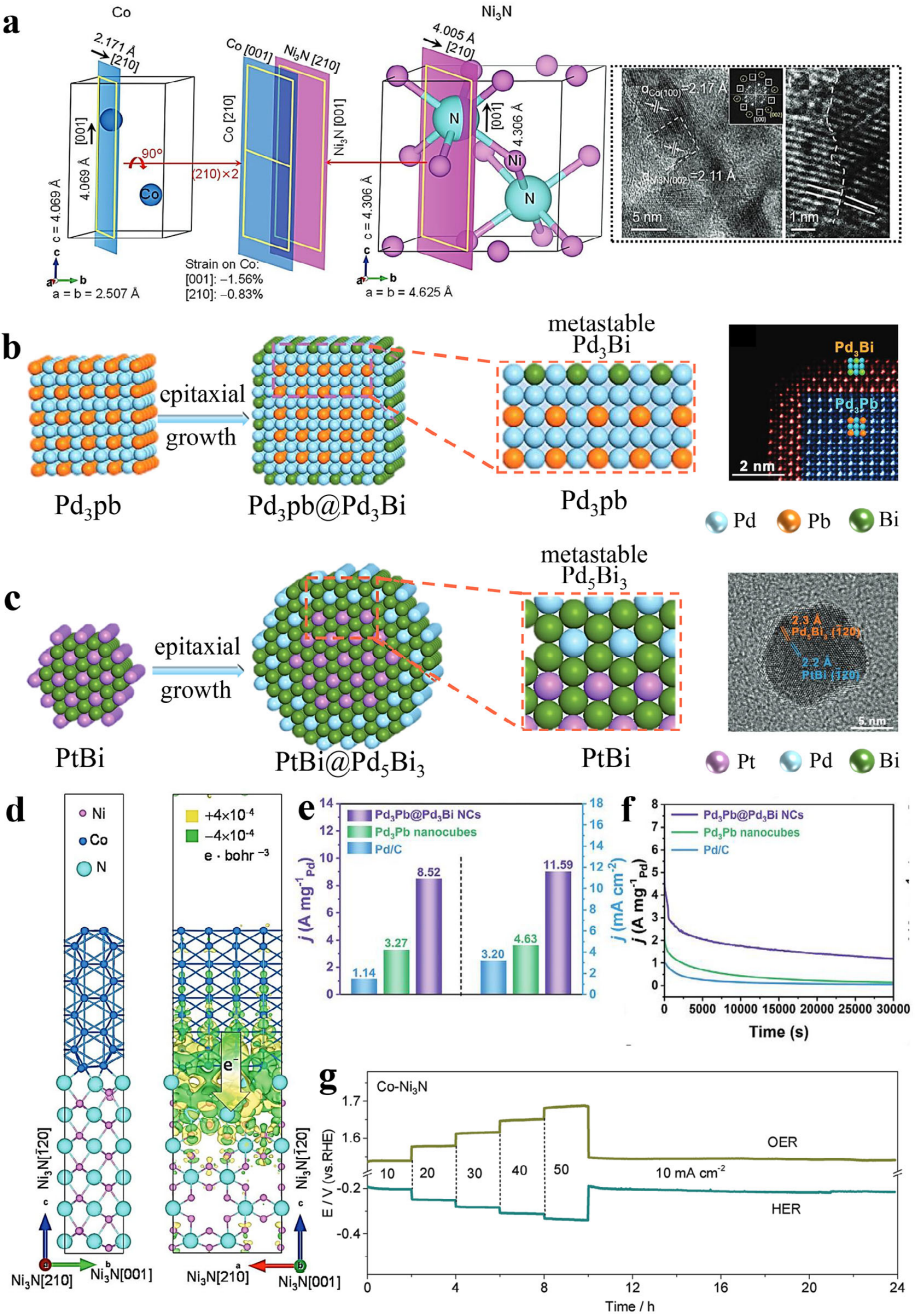
(a) Schematic illustration depicting the lattice matching between metallic Co and Ni_3_N, accompanied by HRTEM images of Co‐Ni_3_N nanorods [91]. (b) Schematic representation of the epitaxial growth from Pd_3_Pb nanocubes to Pd_3_Pb@Pd_3_Bi nanocubes (NCs), with a color image highlighting the two segments: Pd_3_Pb (blue) and Pd_3_Bi (red) [92]. (c) Schematic depiction of the epitaxial growth process from PtBi nanoplatelets (NPLs) to PtBi@Pd_5_Bi_3_ NCs, including the corresponding FFT pattern of an individual PtBi@Pd_5_Bi_3_ NC [92] Copyright 2024, John Wiley and Sons. (d) First‐principles calculations illustrating the charge transfer at the interface [91]. (e) Histogram comparing the mass and specific activities of various catalysts [92]. (f) Current‐time (*i*–*t*) test results for the assessment of different catalysts' performance [92]. (g) Stability tests for Co–Ni_3_N conducted at varying current densities for both HER and OER processes [91] Copyright 2018, John Wiley and Sons.

In addition to metal substrates, semiconductors can also act as epitaxial supports. Zhang et al. prepared a lattice‑matched Mo_2_C–Mo_2_N heterostructure via a gradient‑heating epitaxial route followed by NH_3_ treatment (Figure 3b) [89]. Relative to Pt/C, the heterostructure shows lower overpotentials and higher HER activity in both acidic and alkaline media, and long‑term amperometry and LSV indicate strong stability and activity (Figure 3f). This provides a practical route for lattice‑matched transition‑metal carbide–nitride heterostructures and a path toward high‑performance, low‑cost electrocatalysts [90]. A self‑templated epitaxial has been demonstrated in the controlled synthesis of Co–Fe Prussian blue analogue (PBA) cage‑like, framework, and box structures (Figure 3c); by tuning growth kinetics, the topological complexity was expanded, here, “topology” denotes the internal connectivity, open‐cavity/porous architecture, and cavity geometry of the structures, and the framework architecture exhibited higher catalytic activity, as evidenced by lower overpotentials and smaller Tafel slopes under identical conditions. This work links topology to catalytic behavior, suggesting general strategies to access shape‑ and topology‑dependent activity. Zhu et al. [91] achieved atomic‑scale heteroepitaxial growth of Co on Ni_3_N nanowires: hydrothermally grown Ni_3_N nanowires were annealed in NH_3_ to yield a Co─Ni_3_N heterostructure. First‑principles calculations reveal lattice alignment between hexagonal Co and Ni_3_N (Figure 4a), and charge‑density differences indicate electron transfer from Co to Ni_3_N—with interfacial Ni cations gaining and Co cations losing electrons (Figure 4d). Electrode durability was evaluated by stepping the current density (10, 20, 30, 40, and 50 mA cm^−2^) in sequence for 10 h, confirming robust performance (Figure 4g). This epitaxial architecture promotes nanoscale interdomain electron transfer and significantly boosts catalytic activity.

In contrast to in‑situ epitaxial growth, Han et al. [92] developed a wet‑chemical epitaxial strategy to synthesize metastable Pd–Bi intermetallic nanocrystals (NCs). As shown in Figure 4b, this approach produced transformable Ge–Pd_3_Bi intermetallic nanocrystals and can be extended to other hexagonal intermetallics. For example, metastable Pd_5_Bi_3_ NCs were epitaxially grown on hexagonal close packing PtBi nanoplates (P63/mmc) (Figure 4c). Pd_3_Pb@Pd_3_Bi NCs exhibit a specific activity (SA) of 11.59 mA cm^−2^—2.5× and 3.6× that of Pd_3_Pb nanotubes and Pd/C, respectively (Figure 4e)—and maintain a mass activity (MA) of 1.19 A mg^‑1^Pd after 30 000 s at 0.66 V vs. RHE, far exceeding Pd_3_Pb nanocubes (0.14 A mg^−1^Pd) and commercial Pd/C (0.06 A mg^−1^Pd) (Figure 4f). Overall, the MA and SA are 7.5× and 3.6× those of Pd/C, respectively, with superior stability, advancing the design of high‑performance metastable IMCs for ethanol oxidation and related applications.

Despite its promise, epitaxial growth in electrocatalysis faces several challenges. First, the need for elevated temperatures and vacuum can drive up equipment cost and complexity, limiting scalability. Second, achieving uniform epitaxy over large areas remains difficult, as stringent control of growth conditions is required to ensure structural consistency. Third, interfacial defects or unintended heterostructures formed during growth may compromise catalytic activity and stability. Future work should therefore focus on optimizing growth windows, lowering environmental/operational hazards, and exploring new epitaxial strategies that enable efficient, uniform growth, thereby accelerating the deployment of electrocatalytic materials in energy‑conversion technologies.

### 3.2 Heterostructure Coating/Array

**FIGURE 5:**
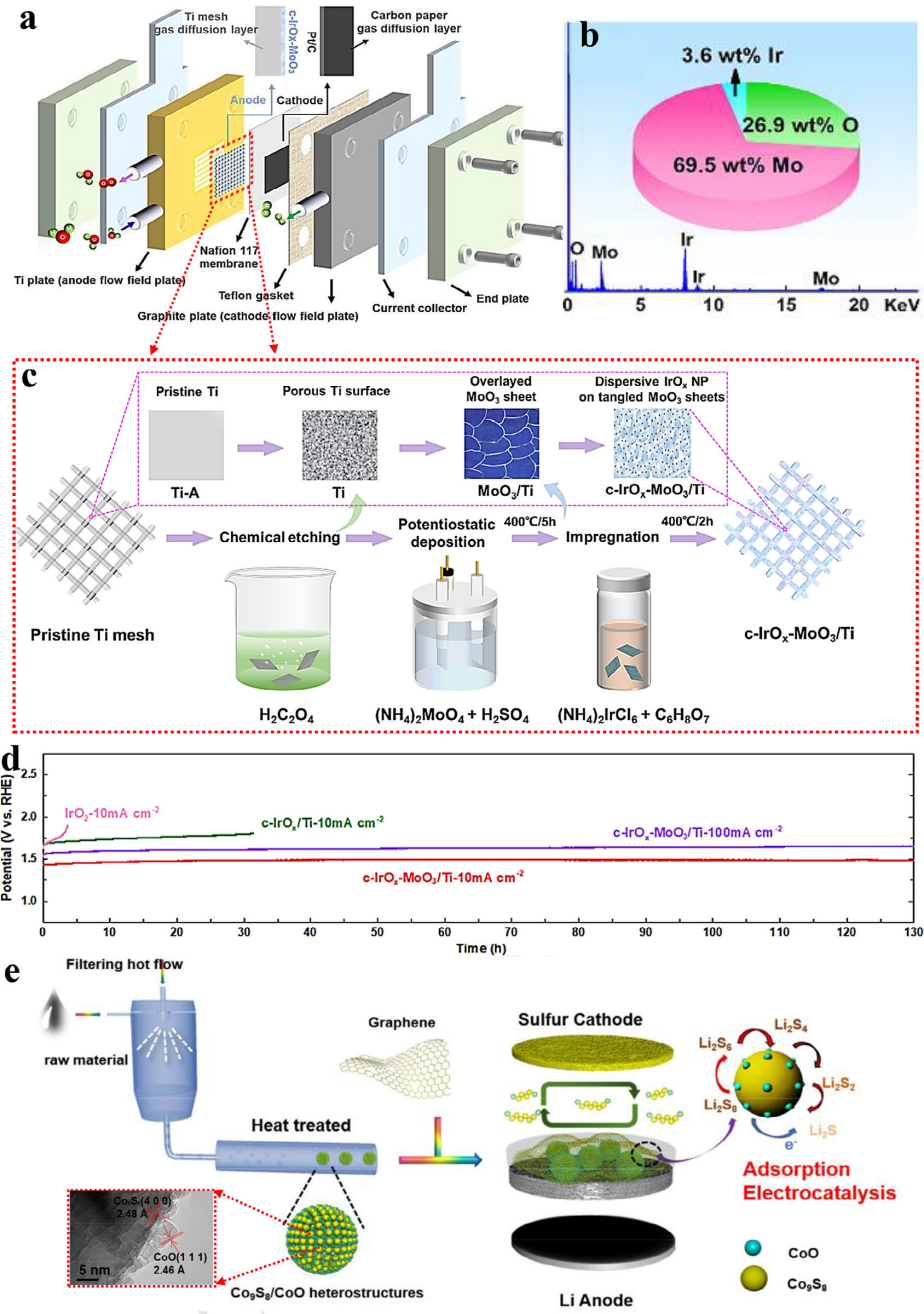
(a) Schematic diagram of a PEM electrolyzer. (b) STEM‐EDS spectra with an inserted pie chart representing the corresponding elemental content based on the EDS spectra, obtained from c‐IrOx‐MoO_3_/Ti. (c) Schematic illustration depicting the fabrication process of the c‐IrOx‐MoO_3_/Ti heterocomplex. (d) Chronopotentiometry curves for the as‐prepared electrocatalysts [95]. Copyright 2023, Elsevier. (e) Illustration of the synthesis process for Co_9_S_8_/CoO heterojunctions and the configuration of a Li‐S battery with a separator modified by the Co_9_S_8_/CoO‐graphene composite layer [96]. Copyright 2019, Elsevier.

**FIGURE 6:**
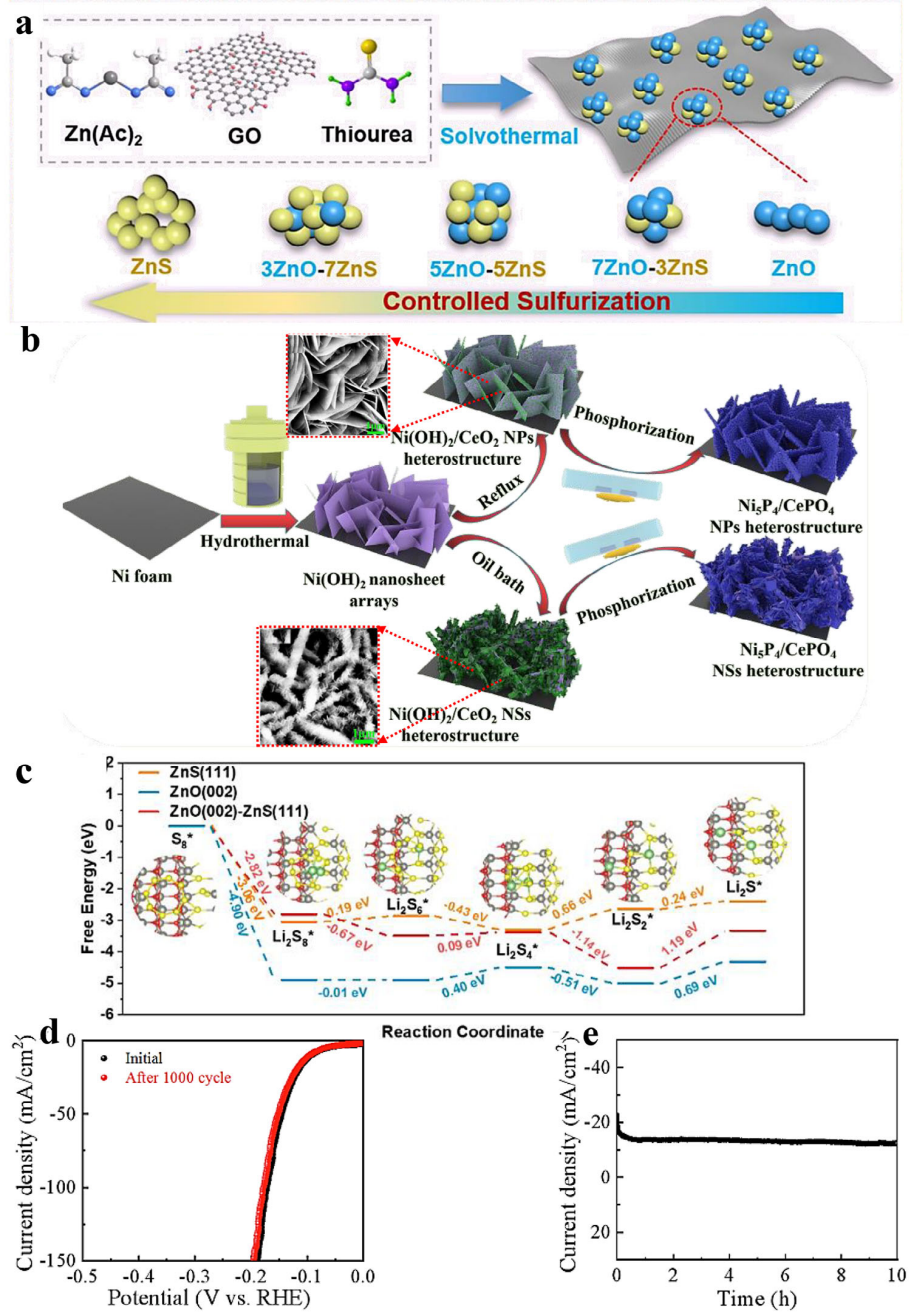
(a) Schematic illustration depicting the formation of ZnO‐ZnS/rGO heterostructures with varying sulfurization degrees [97]. (b) Schematic depiction of the preparation process for Ni_5_P_4_/CePO_4_ core/shell heterostructure arrays, along with SEM images [98]. (c) Free energy changes during the multistep reduction of sulfur species on ZnS(111), ZnO(002), and ZnO(002)‐ZnS(111) surfaces [97]. Copyright 2022, Elsevier. (d) HER LSV (Linear Sweep Voltammetry) curves of Ni_5_P_4_/CePO_4_ NPs (Nanoparticles) heterostructure array initially and after 1000 cyclic voltammetry cycles. (e) Time‐dependent HER current density curve of Ni_5_P_4_/CePO_4_ NPs heterostructure array for 10 h [98]. Copyright 2024, Elsevier.

Heterostructure coating/array denotes conformal or patterned deposition of one or more catalytic phases on conductive scaffolds (e.g., Ni foam, carbon cloth) without requiring lattice matching. This approach emphasizes architectural and interfacial control—film thickness/coverage and array spacing/orientation/areal density—to maximize the electrochemically accessible surface area (ECSA), ensure intimate electrical contact, and enhance durability under bias. Depositing active phases as coatings or oriented arrays improves dispersion and site accessibility while providing efficient electron/ion pathways [93, 94]. For example, Chen et al. [95] eletrodeposited an IrOx–MoO_3_ composite catalyst on a titanium mesh, followed by citric acid‐assisted impregnation–calcination process. Figure 5a,c outlines the schematic synthesis and its integration in a proton exchange membrane electrolyzer, while Figure 5b shows an ultralow Ir loading (∼28.35 µgIr cm^−2^), markedly below that of IrOx/Ti and commercial IrO_2_. The resulting c‐IrOx–MoO_3_/Ti catalyst exhibited an overpotential of ≈200 mV at 10 mA cm^−^^2^ and sustained operation for >130 h at 100 mA cm^−^^2^ in acidic OER (Figure 5d). In another work, coating separators with a Co_9_S_8_/CoO heterostructure (Figure 5e) improved both electrocatalytic performance and long‑term stability in Li–S batteries, highlighting the broader utility of heterointerfaces in energy systems [96]. Material selection is pivotal when designing such coatings. To simultaneously achieve strong adsorption and rapid conversion of lithium polysulfides (LiPSs) in Li–S batteries, a ZnO–ZnS/rGO heterostructure was engineered as a separator coating [97]. Figure 6a depicts the synthesis route. By tuning the sulfidation degree, a ZnO:ZnS ratio of 7:3 achieved the best overall performance by balancing adsorption strength with catalytic turnover. Calculated Gibbs free‑energy changes (Figure 6c) for the final three reduction steps (Li_2_S_6_ → Li_2_S_4_ → Li_2_S_2_ → Li_2_S) are 0.14 eV on the ZnO(002)–ZnS(111) heterointerface, smaller than on ZnO(002) (0.58 eV) or ZnS(111) (0.47 eV); the Li_2_S decomposition barrier is likewise lower (0.36 eV vs. 0.81/0.53 eV). These results indicate that the ZnO–ZnS heterointerface better facilitates LiPS‐to‐Li_2_S conversion., underscoring how interfacial design can reconcile competing requirements for adsorption and catalysis.

Heterostructure arrays are built by organizing multiple components into ordered nano‑architectures on a substrate, enabling precise control of size, geometry, and spacing to regulate transport and kinetics. Liang et al. [98] fabricated a crystalline Ni_5_P_4_ nanosheet/amorphous CePO_4_ nanocrystal core–shell array (Figure 6b) for overall water splitting (OWS). In alkaline media, the array required only 94 mV (HER) and 191 mV (OER) to reach 10 mA cm^−2^. A symmetric two‑electrode device using this array achieved 10 mA cm^−2^ at 1.535 V. Durability was confirmed by an unchanged HER polarization curve after 1000 cycles (Figure 6d) and stable OER current over 10 h (Figure 6e), indicating excellent cycling stability and interfacial robustness.

The progress of heterostructure coatings/arrays hinges on controlling film thickness/coverage and array pitch/orientation on 3D scaffolds to raise ECSA without pore flooding, securing adhesion‐ and corrosion‐resistant coating–substrate contacts verified by adhesion metrics and R_ct_ retention under bias/flow, and co‐designing electron/ion pathways to avoid diffusion bottlenecks at high areal loading. Addressing these issues through interdisciplinary efforts—combining advanced synthesis, operando characterization, modeling, and device‑level engineering—will allow heterostructure coating/array technologies to deliver step‑changes in electrocatalytic performance for sustainable energy applications.

### 3.3 In Situ Growth

**FIGURE 7:**
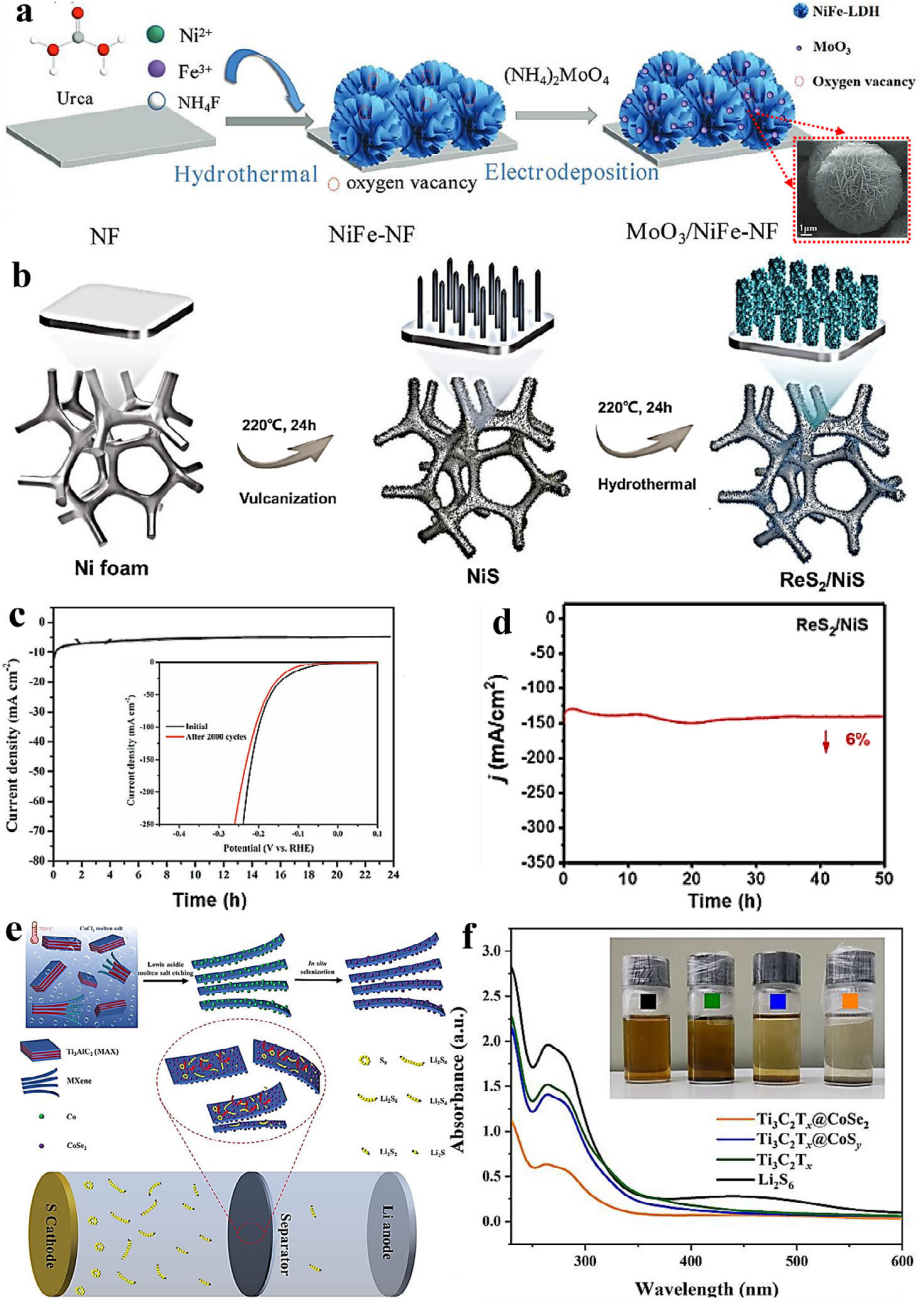
(a) Schematic representation of the synthesis process of MoO_3_/NiFe‐NF and SEM images showcasing MoO_3_/NiFe‐NF [102]. Copyright 2023, John Wiley and Sons. (b) Schematic illustration detailing the fabrication of heterostructured ReS_2_/NiS composites [104]. (c) Long‐term stability assessment of MoO_3_/NiFe/NF [102]. (d) Electrochemical stability evaluation of ReS_2_/NiS measured at 150 mA cm^−2^ for 50 h [104]. Copyright 2023, Elsevier. (e) Schematic depiction of the synthesis process of Ti_3_C_2_T_x_@CoSe_2_ heterostructure and the configuration of Li‐S battery utilizing the modified separator [106]. (f) Image of Li_2_S_6_ solution after adsorption with the prepared samples alongside the corresponding UV‐vis absorption spectra of the solution after 12 h. Additionally, the XPS spectrum of the Ti_3_C_2_T_x_@CoSe_2_ heterostructure is provided before and after Li_2_S_6_ adsorption [106]. Copyright 2022, Elsevier.

**FIGURE 8:**
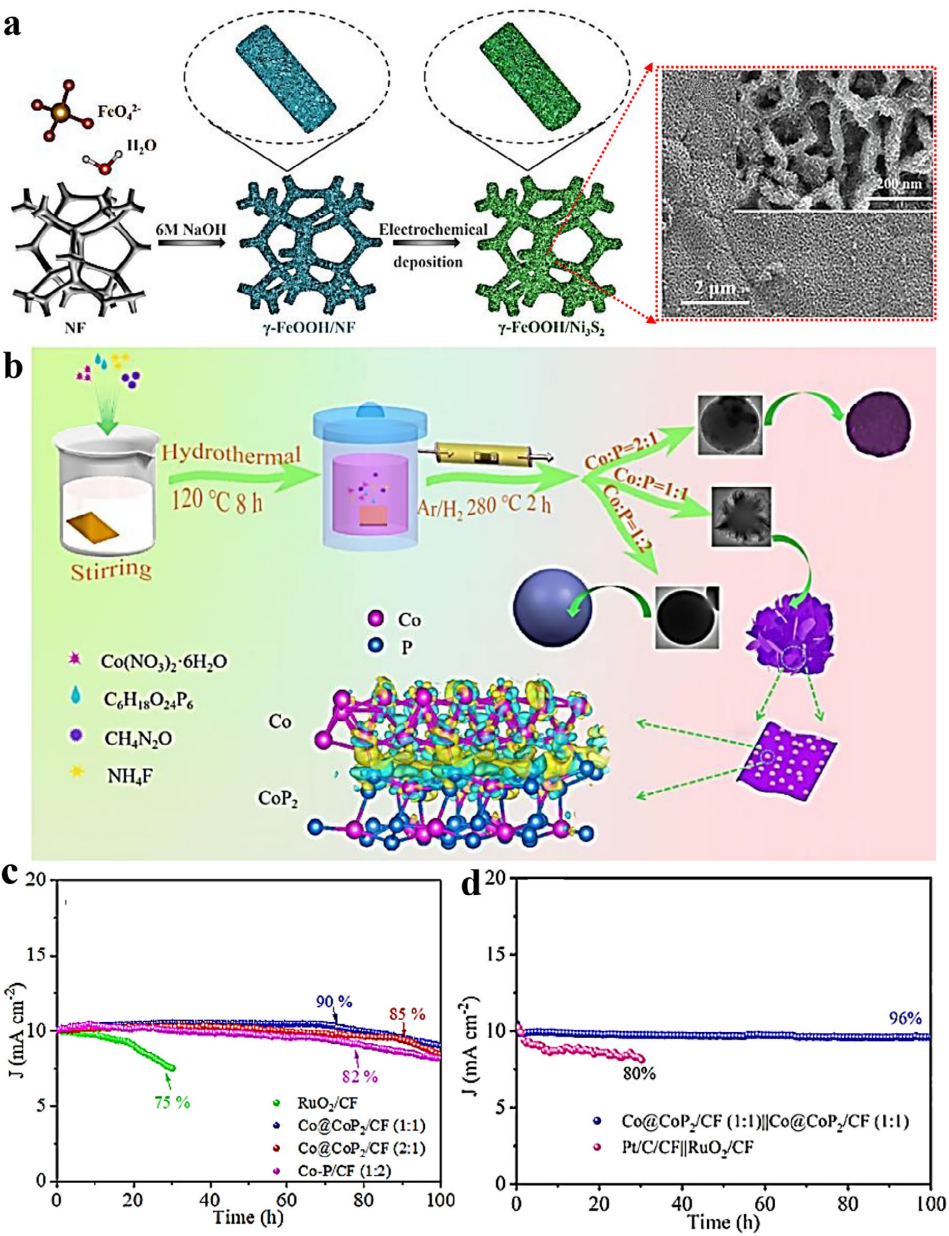
(a) Schematic depiction of the preparation process for c‐FeOOH/Ni_3_S_2_ and SEM images showcasing c‐FeOOH/Ni_3_S_2_ [105]. Copyright 2023, Elsevier. (b) Schematic representation of the formation process of the three Co‐P composites [103]. (c) Time‐dependent current density curves of Co@CoP_2_/CF (2:1), Co@CoP_2_/CF (1:1), Co‐P/CF (1:2), and RuO_2_/CF at a fixed overpotential for 100 h [103]. (d) Long‐term stability assessment conducted at a constant voltage [103]. Copyright 2023, Elsevier.

In situ growth plays a pivotal role in electrocatalytic heterostructure constructing by harnessing the local electrochemical environment to direct catalyst formation [99, 100, 101]. By judiciously tuning surface potential or pH, precursors can be deposited and/or transformed on the working electrode (conductive substrate) at designated regions, without additional soft/hard sacrificial templates or post‐growth transfers, thereby constructing ordered, robust active sites on electrode surfaces. As a representative example, MoO_3_ was immobilized on nickel–iron layered double hydroxide (NiFe‐LDH) via electrodeposition under mild conditions, underscoring the practical utility of in situ growth [102]. The electrode retains its current density under a constant 10 mA cm^−^^2^ load for 24 h (Figure 7c) and, when employed as both anode and cathode in OWS, delivers 10 mA cm^−^^2^ at a low cell voltage of 1.43 V—surpassing systems based on commercial Pt/C and IrO_2_. In a two‑step route, Co–P composites were grown on carbon fiber by first forming precursors from urea, cobalt nitrate, ammonium fluoride, and phytate (120 °C, 8 h), followed by conversion in Ar/H_2_ at 280 °C [103]. Among the series, nanoflower‑like Co@CoP_2_ exhibits excellent corrosion resistance and stability in durability tests (Figure 8c,d). Surface‑confined growth has also yielded a ReS_2_/NiS heterostructure on nickel foam (NF) [104]. Metallic Ni reacts with thiourea to form NiS nanowires in situ; subsequent hydrothermal growth of ReS_2_ nanosheets on these nanowires generates dense heterointerfaces (Figure 7b). The resulting ReS_2_/NiS@NF catalyst achieves an overpotential of 78 mV at 10 mA cm^−^^2^ for HER and sustains 150 mA cm^−^^2^ over an extended operation duration (Figure 7d), highlighting the role of multi‑component synergy in accelerating water dissociation and hydrogen desorption.

Using NF as a scaffold, a three‑dimensional c‑FeOOH/Ni_3_S_2_ architecture was prepared by forming Fe(OH)_3_ colloids in situ (from FeO_4_^2^^−^ and H_2_O), coagulating and transforming them into c‑FeOOH nanosheets under NaOH, and then electrodepositing Ni_3_S_2_ nanosheets atop the oxide layer [105]. constructed a three‑dimensional, hierarchically structured c‑FeOOH/Ni_3_S_2_ heterostructure (Figure 8a). The interpenetrating 3D heterostructure furnishes abundant, strongly coupled active sites for bifunctional electrocatalysis (Figure 8a). Beyond water splitting, in situ growth is effective in energy‑storage catalysis. Cai et al. anchored CoSe_2_ nanoparticles strongly onto Ti_3_C_2_T_x_ MXene via Lewis‑acid molten‑salt etching followed by in situ selenization, forming Ti_3_C_2_T_x_@CoSe_2_ (Figure 7e) [106]. Visual adsorption and UV–vis tests (Figure 7f) show enhanced Li_2_S_6_ capture, indicating more active sites for LiPSs conversion and offering a broadly applicable route to regulate shuttle suppression in Li–S batteries.

Despite these advances, several issues remain. (i) Process control and mechanistic insight: achieving nanoscale precision over nucleation/growth and clarifying the dynamic reconstruction of interfaces under electric bias demand improved operando diagnostics and modeling. (ii) Long‑term stability: maintaining interfacial integrity against corrosion, phase transformation, and mechanical detachment in realistic electrolytes and high‑current operation is critical. (iii) Scalability and cost: translating laboratory protocols to uniform, large‑area, high‑throughput manufacturing while minimizing energy/precursor costs is a key practical consideration. Addressing these challenges will enable in situ growth to deliver a finely engineered, durable heterojunction for practical electrocatalysis.

### 3.4 Solvothermal Method

**FIGURE 9:**
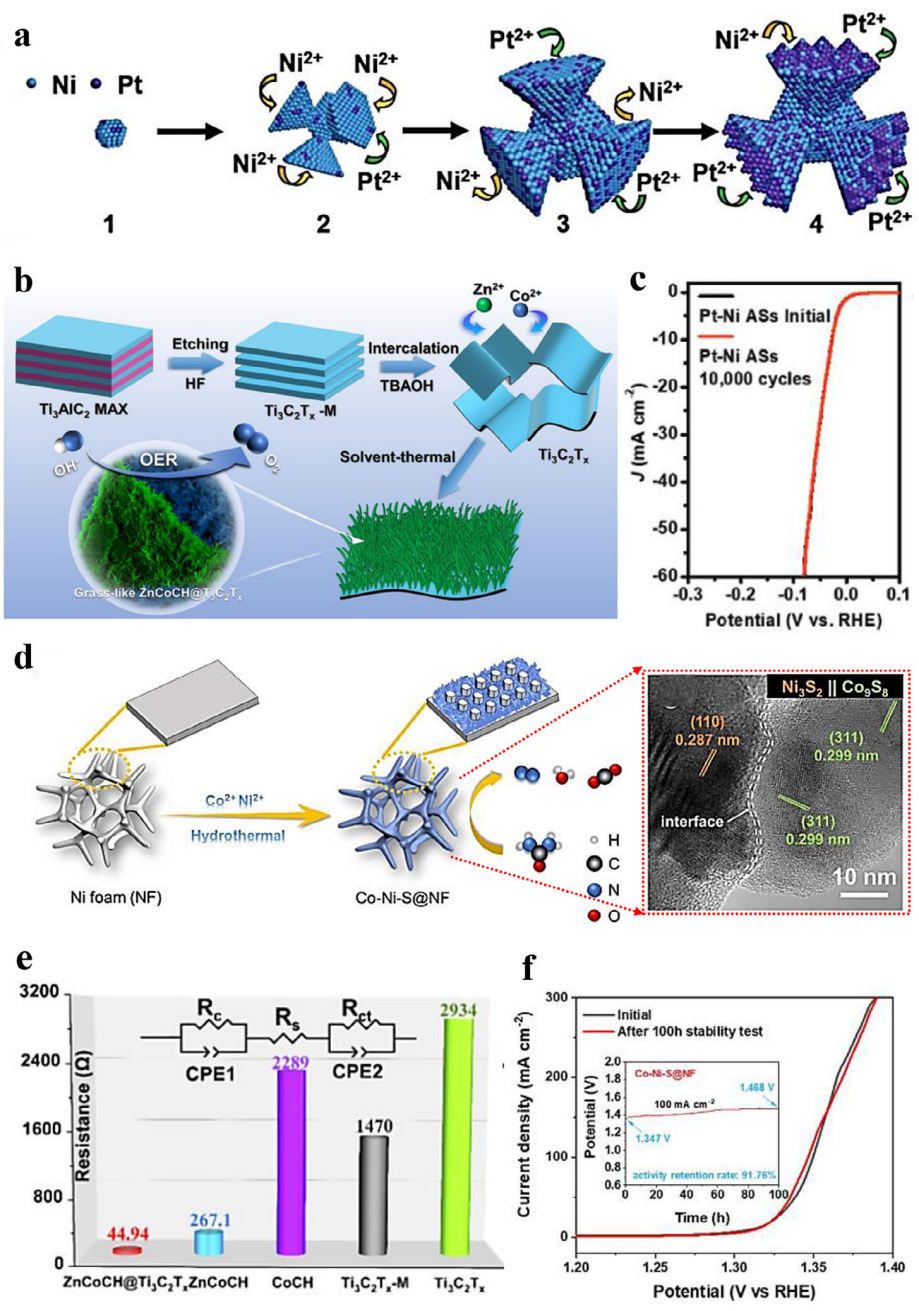
(a) Schematic depiction of the preparation process for Pt‐Ni alloy structures [111]. (b) Schematic illustration of the grass‐like ZnCoCH@Ti_3_C_2_T_x_ heterostructure and its application as an OER electrode [112]. (c) Durability assessment of Pt‐Ni alloy structures. The polarization curves were recorded before and after 10,000 potential cycles in 1.0 M KOH aqueous solution from 0.1 to ‐0.1 V (vs. RHE) [111]. Copyright 2018, John Wiley and Sons. (d) Schematic illustration of the formation of heterogeneous Co‐NiS@NF and HRTEM image showing a clear interface between the (110) facet of Ni_3_S_2_ and the (311) facet of Co_9_S_8_ [113]. (e) The charge transfer resistance (R_ct_) applied to calculate the bilayer capacitance (Cdl) of the above samples [112]. Copyright 2023, Elsevier. (f) LSV curves of Co‐Ni‐S@NF before and after a 100‐h stability test. The inset shows the chronoamperometric curve at a constant current density of 100 mA cm^−2^ [113]. Copyright 2022, Royal Society of Chemistry.

**FIGURE 10:**
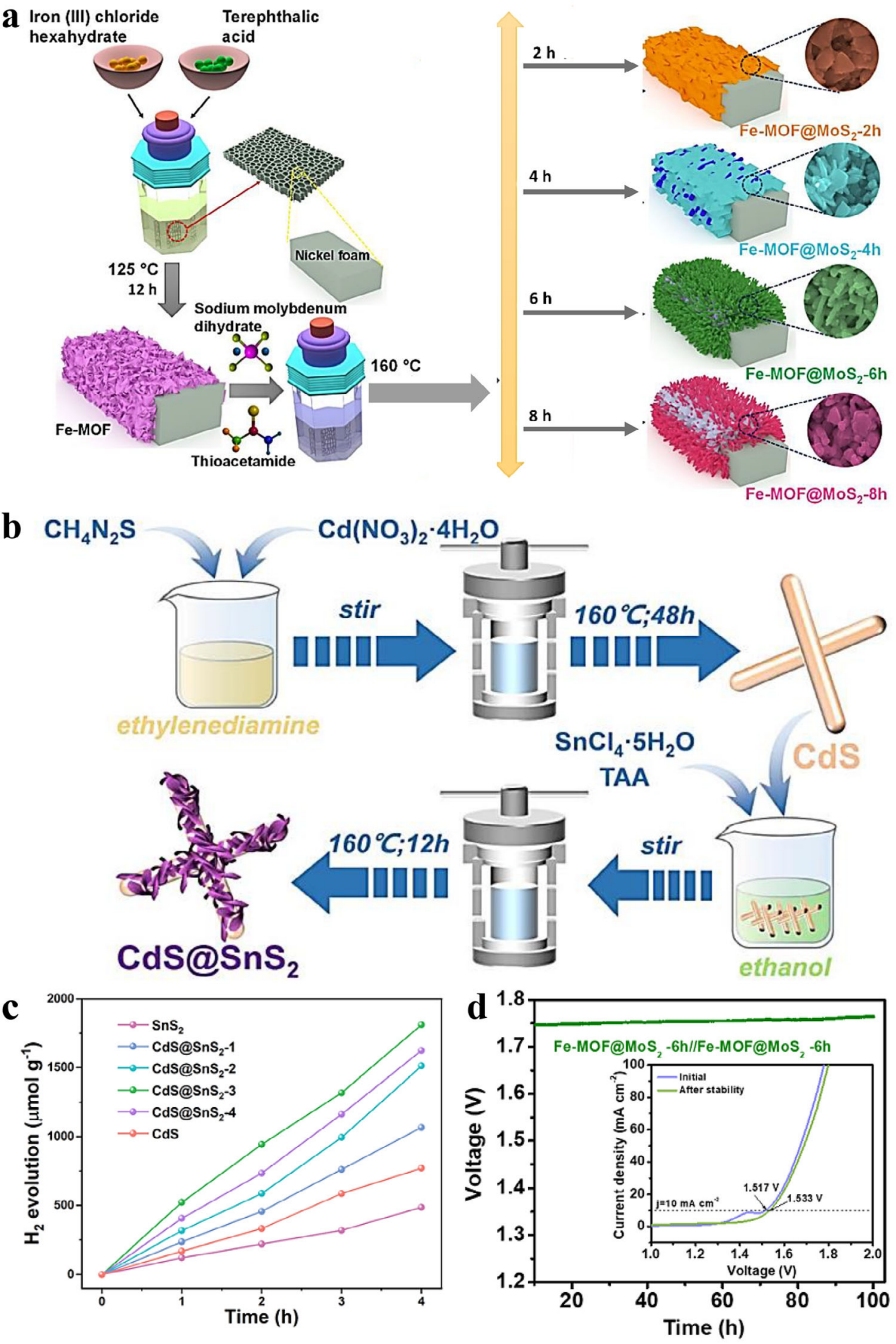
(a) Schematic illustration depicting the straightforward synthesis process for Fe‐MOF and the heterostructures Fe‐MOF@MoS_2_‐2 h, Fe‐MOF@MoS_2_‐4 h, Fe‐MOF@MoS_2_‐6 h, and Fe‐MOF@MoS_2_‐8 h on NF (nickel foam) substrates [114]. (b) Schematic representation for the fabrication of the 1D/2D core/shell CdS@SnS_2_ composites [115]. (c) Time‐dependent piezocatalytic hydrogen evolution performance under ultrasonic vibration over the as‐prepared piezocatalysts [115]. Copyright 2023, Royal Society of Chemistry. (d) Chronopotentiometric durability study of the Fe‐MOF@MoS_2_‐6 h//Fe‐MOF@MoS_2_‐6 h electrolyzer at a current density of 50 mA cm^−2^ for 100 h (inset shows linear sweep voltammetry (LSV) polarization curves before and after the stability test) [114]. Copyright 2023, Elsevier.

The solvothermal method is a synthetic approach in which reactions are carried out in sealed vessels using non‐aqueous organic solvents or mixed solvent systems composed of water and organic solvents at elevated temperature and pressure [107, 108]. In this review, solvothermal synthesis is therefore not restricted to strictly anhydrous media, and many reported solvothermal reactions are also conducted in water/organic mixed solvents to regulate precursor solubility, nucleation and growth behavior, and catalyst structure [109, 110]. Compared to the traditional hydrothermal method, the solvothermal method provides a more flexible and tunable synthesis environment, particularly for water‐sensitive materials, and enables precise control over the morphology, size, and structure of materials by adjusting the reaction conditions. Under solvothermal conditions, the physical and chemical properties of the solvent system (including pure organic solvents and water/organic mixed solvents), such as density, viscosity, and solute solubility, can differ markedly from those under ambient conditions, offering opportunities to synthesize materials with special morphologies and structures. This section discusses the solvothermal method from two aspects: a one‐pot solvothermal method and a two‐step solvothermal method. The one‐pot solvothermal method involves adding all reactants and solvents to a high‐pressure reaction vessel at one time and conducting the reaction under certain temperature and pressure. This method is favored by researchers for its simplicity and ease of control. One‐pot solvothermal method has been successfully applied to the preparation of various electrocatalysts. For example, a study synthesized well‐crystallized lotus‐bud‐like Pt‐Ni alloy superstructures (ASs) through a simple one‐pot solvothermal method [111]. The specific process involved the initial formation of nickel‐rich polyhedral nanostructures. In such syntheses, formaldehyde, as one of the reactants, decomposes into CO and H_2_ at elevated temperatures. The oleylamine solvent and as‐produced H_2_ promote the selective reduction of Ni^2+^ ions, even though the standard Ni^2^^+^/Ni potential (–0.25 V vs. SHE) is relatively negative. Subsequently, the polyhedral nickel‐rich nanostructures transformed into branched structures, possibly originating from the polymorphism of nickel. With an increase in the reaction time, the branched nanostructures became larger (Figure 9a). These unique Pt‐Ni ASs exhibited excellent electrocatalytic activity and stability under alkaline conditions, better than commercial Pt/C. The overpotential obtained for OER was only 27.7 mV at a current density of 10 mA cm^−2^, and the turnover frequency reached 18.63 H_2_ s^−1^ at an overpotential of 50 mV. Additionally, the durability test of Pt‐Ni ASs also indicated that the catalyst has good stability and durability (Figure 9c). This study synthesized superstructures with spatial heterogeneity through a one‐pot solvothermal method, promoting their promising applications in catalytic reactions. Another study synthesized a ZnCoCH@Ti_3_C_2_T_x_ heterostructure through the solvothermal method [112], where unique grass‐like zinc‐doped double metal cyanide hydroxide grew in situ on the surface of MXene (Figure 9b). Experiments and DFT calculations showed that MXene played a dual‐enhancement role in improving OER performance, acting as an electron‐conduction path and a reductant. As a conductive carrier, MXene alleviated the aggregation of ZnCoCH, and Figure 9e shows that ZnCoCH@Ti_3_C_2_T_x_ has the smallest Rct and the fastest charge transfer rate among the controls. As a reductant, MXene provided more redox sites by reducing ZnCoCH and promoted the adsorption of intermediate species on the catalyst. This study proposes a reasonable design idea for MXene–transition‐metal heterostructure OER catalysts. Xu et al. [113]. synthesized a heterostructured cobalt and nickel‐based (Co–Ni–S@NF) electrocatalyst anchored on nickel foam through a one‐pot solvothermal method, and Figure 9d presents the formation process of Co–Ni–S@NF and its corresponding HRTEM image. Figure 9f shows the LSV curves collected before and after a 100 h constant‐current UOR durability test at 100 mA cm^−^^2^ in 1.0 M KOH + 0.33 M urea; the inset chronogram confirms stable operation throughout. This work offers a one‐pot solvothermal route to efficient, low‐cost urea electro‐oxidation catalysts. The method features simplicity, short reaction times, and tunable composition/stoichiometry and coverage (via precursor ratios, temperature, and time), enabling quick construction of heterointerfaces. However, it remains limited in the precise control of morphology and structure—for example, facet/orientation selection, domain (shell) thickness, and defect distribution. In contrast, the two‐step solvothermal method can achieve more precise control over the morphology, size, and structure of the electrocatalyst, thereby optimizing its electrocatalytic performance. The two‐step solvothermal method is an improved solvothermal synthesis strategy that controls the material formation process by running reactions in stages with different reactants/solvents. Initially, a preliminary solvothermal reaction is carried out to form intermediate products, and then additional solvents and reactants are added in the latter step to further regulate the material's morphology and structure. This method has shown great potential in preparing electrocatalysts with complex structures and excellent performance. For example, a Fe‐MOF@MoS_2_ hybrid architecture was prepared through a two‐step solvothermal method. As shown in Figure 10a, Fe‐MOF was first in situ grown on NFs using terephthalic acid and FeCl_3_·6H_2_O. Subsequently, MoS_2_ was in situ grown on Fe‐MOF for different reaction times, resulting in different products (named Fe‐MOF@MoS_2_‐2 h/4 h/6 h/8 h). Tuning the growth time adjusts the MoS_2_ coverage and morphology, thereby increasing the number of electroactive sites and improving electrocatalytic activity. Before and after a 100‐h chronopotentiometry durability test at 100 mA cm^−^^2^ in 1.0 M KOH (HER), LSV polarization curves were collected, and only a slight positive shift in overpotential and no NF oxidation peak (Figure 10d), indicating that the symmetric electrolyzer using Fe‐MOF@MoS_2_‐6 h on both electrodes maintains high durability in alkaline electrolyte. Another study prepared CdS/SnS_2_ composite materials through a two‐step solvothermal method [115]. First, the pre‐synthesized CdS precursor samples were mixed with absolute ethanol and subjected to ultrasonic stirring, then SnCl_4_ **^.^** 5H_2_O and thioacetamide were added, and then reacted under constant temperature conditions. The solvothermal reaction afforded CdS@SnS_2__−__x_ composites (Figure 10b). The experimentally measured piezoelectrocatalytic hydrogen production rate and Rhodamine B degradation rate of CdS@SnS_2_ reached 3.7 times and 7.4 times that of pure SnS_2_, and 2.4 times and 4.6 times that of pure CdS. In addition, under piezo‐electrocatalytic water splitting (ultrasonic irradiation), all samples generated H_2_, and the H_2_ yield increased with irradiation time (Figure 10c), indicating that the CdS/SnS_2_ heterojunction also supports mechano‐electro coupling. This study constructs a CdS/SnS_2_ heterojunction piezo‐electrocatalyst via a two‐step solvothermal route and evaluates its piezo‐electrocatalytic H_2_ production performance.

Solvothermal methods face the following challenges. First, the selection of solvents and optimization of reaction conditions need further research to achieve a more efficient and environmentally friendly synthesis process. Second, for the synthesized electrocatalysts, performance under operating conditions should be rigorously assessed—covering stability, durability, and long‐term activity. In addition, large‐scale production and cost‐effectiveness are also important factors to consider in future research. The future development direction will focus on the following aspects: first, developing new solvents and additives to achieve more precise material synthesis; second, combining advanced characterization techniques and theoretical calculations to resolve the formation mechanism and performance optimization pathways of electrocatalysts; third, exploring the combination of the solvothermal method with other synthetic methods to prepare electrocatalysts with complex structures and excellent performance.

### 3.5 Hybrid Synthetic Strategies

Many high‑performing heterostructured electrocatalysts are prepared through sequential, hybrid workflows rather than a single synthetic route. A key advantage of this strategy is that distinct functions can be effectively decoupled: an initial step establishes a mechanically robust and electronically percolating scaffold or primary phase with a well‑defined morphology, while a subsequent step introduces a secondary component with tunable thickness and coverage, as well as strong interfacial adhesion [116]. This stepwise design increases the density and connectivity of heterointerfaces and offers additional freedom to regulate nucleation and growth, interfacial coupling, and junction continuity beyond what is typically achievable in one‑pot syntheses. A representative example is the hydrothermal growth followed by the electrodeposition route reported by Wang and co‑workers, where Ni_x_S_y_ nanorod arrays were first constructed on nickel foam and then coated with a conformal MnO_x_H_y_ shell via mild anodic electrodeposition, yielding a three‑dimensional core–shell Ni_x_S_y_@MnO_x_H_y_ architecture [117]. In this design, hydrothermal synthesis defines an open, high‑surface‑area backbone, whereas electrodeposition enables thickness‑ and coverage‑controlled shell formation that maximizes interfacial contact while maintaining intimate electrical coupling. The resulting interfacial robustness and shell‑assisted stabilization translate into durable bifunctional activity for the hydrogen and oxygen evolution reactions, supporting stable overall alkaline water splitting.

More broadly, hybrid syntheses in heterostructure and interface engineering often follow two recurring patterns. One couples a morphology‑defining growth step with a conformal deposition step to maximize electrically connected interface density. The other couples an initial assembly or growth step with a subsequent conversion step to generate the target phase junction while preserving the parent architecture; typical conversions include phosphidation, selenization, nitridation, carburization, and controlled oxidation or reduction. While these multistep routes substantially broaden the accessible interface architectures, they also complicate mechanistic attribution, because improvements in apparent activity may arise from concurrent changes in surface area, porosity, defect density, or mass transport. Accordingly, hybrid‑route studies should clarify the role of each step and quantify how it alters interfacial and kinetic descriptors, such as junction coverage, shell thickness, electrochemically active surface area (ECSA), and charge‑transfer resistance (R_ct_), while benchmarking against well‑matched single‑phase and single‑step controls. From an engineering perspective, preserving interfacial integrity during the final conversion or activation step is essential; thermal budgets and chemical environments should be selected to minimize interdiffusion, delamination, and pore blockage so that electrically connected interfaces and high site utilization are retained under device‑relevant current densities.

## 4 Application of Heterostructured Catalysts

Interfacial engineering is a core strategy for boosting electrocatalytic performance and has been widely applied across different reactions. The advantages of heterostructured catalysts include [118, 119, 120, 121, 122, 123]: (1) strong interfacial electronic coupling increases electronic conductivity across the junction—experimentally reflected by lower R_ct_ and, in turn, higher exchange current densities under operation; (2) controlled band alignment at the near‑surface junction modulates the surface electronic structure and adsorption energetics of key intermediates, improving intrinsic kinetics (e.g., more favorable onset/half‑wave potentials, smaller Tafel slopes) without conflating them with geometric area effects; (3) dual‑site/perimeter chemistry emerges at the junction, where complementary sites (for example, an oxophilic site for H_2_O activation paired with a metal site for H adsorption) cooperatively accelerate the rate‑determining elementary steps; and (4) strengthened interfacial bonding and confined reconstruction enhance durability, helping retain ECSA and R_ct_ under prolonged bias/flow. The following sections present selected examples of heterostructured catalysts in electrochemical energy conversion and storage, illustrating how heterostructure constructing can markedly enhance performance. To ensure timeliness and relevance, we emphasize literature published after 2020.

### 4.1 Oxygen Reduction Reaction (ORR) and Oxygen Evolution Reaction (OER)

The oxygen reduction reaction (ORR) underpins fuel cells, green H_2_O_2_ synthesis, and metal–air batteries, and thus critically determines the performance of these energy‑conversion technologies. Platinum‑based catalysts have been extensively investigated because of their high activity; however, their high cost and scarcity motivate an ongoing search for alternatives [124]. ORR proceeds predominantly via two pathways: either four‑electron (4e^−^) or two‑electron (2e^−^) route. The 4e^−^ pathway—preferred in fuel cells—fully reduces O_2_ to water through sequential steps of O_2_ adsorption, electron transfer, O─O bond scission, protonation/hydration, and intermediate desorption. On catalyst surfaces with low oxygen coverage, O─O cleavage typically precedes *OH formation (a dissociative mechanism). The 2e^−^ route yields H_2_O_2_ and is favored at higher oxygen coverage, where O─O cleavage follows *OOH formation (an associative mechanism) [30]. Heterostructures can markedly enhance ORR by leveraging strong interfacial electronic coupling that optimizes charge transfer and the adsorption energies of key intermediates. For example, Sakaushi and co‑workers activated an otherwise inert Au surface by overlaying it with a corrugated carbon–nitrogen 2D porous framework, enabling efficient four‐electron ORR on Au [125]. Likewise, CoS_2_/Cu_2_S–NF assemblies delivered an excellent power density of 92.06 mW cm^−^^2^ within 1.00–0.23 V, showing an average electron‑transfer number *n* ≈ 3.74 and a minimum H_2_O_2_ yield ≈ 11.21%, consistent with a dominant 4e^−^ pathway [126]. In another illustration, acetate‑assisted carbonization produced hollow, hierarchically porous nitrogen‐doped hard‐porous‐carbon (N‐HPC) heterostructures; Bader charge analysis revealed charge replenishment from the bottom to the top carbon layers, generating more negatively charged top‑layer carbon that facilitates O_2_ adsorption/activation and improves the Gibbs free‑energy profile for the 4e^−^ ORR [127]. Collectively, these studies underscore how precise interface design modulates catalyst electronic structure, furnishes appropriate active sites, and elevates ORR activity. Although the key steps and intermediates of ORR are broadly agreed upon, the catalyst‑ and environment‑dependent details continue to be refined, guiding the design of more efficient ORR catalysts [128]. Table 1 summarizes recent progress in 4e^−^‑ORR catalysts achieved through heterostructure engineering [112, 113, 114, 115, 116, 117, 118, 119, 120, 121, 122, 123, 124, 125, 126, 127, 128, 129, 130, 131, 132, 133, 134, 135, 136, 137, 138, 139, 140, 141, 142, 143, 144, 145, 146, 147, 148, 149, 150, 151, 152, 153, 154]. Here, *E*_onset_ (V vs. RHE) denotes the onset potential and *E*_1/2_ (V vs. RHE) the half‑wave potential; “20 wt% Pt/C” refers to a benchmark catalyst containing 20 mass% Pt supported on carbon. Notably, in many cases, heterostructured catalysts match or surpass commercial Pt/C, highlighting the synergistic enhancement imparted by well‑constructed heterointerfaces. This synergy arises from interfacial electronic‑structure tuning and optimized intermediate adsorption, which together lower activation barriers and accelerate reaction rates [125, 126, 127, 128, 129, 130, 131, 132, 133, 134, 135, 136, 137, 138, 139, 140, 141, 142, 143, 144, 145, 146, 147, 148, 149, 150, 151, 152, 153, 154, 155, 156, 157, 158, 159, 160, 161, 162, 163, 164, 165, 166, 167].

**TABLE 1:** Electrocatalytic performance of representative 2e^−^/4e^−^ ORR catalysts in alkaline media. *E*_o__n__s__e__t_ and *E*_1_⁄_2_ denote the onset and half‑wave potentials, respectively (vs. RHE). Unless otherwise stated, the electrolyte is 0.1 M KOH (1.0 M KOH for CoP/CoN@NCNRS/CC). Data of commercial 20 wt% and Pt/C are included for comparison.

Electrocatalyst	*E*_onset_ (V vs. RHE)	*E*_1/2_ (V vs. RHE)	20 wt% Pt/C		Reference
			*E*_onset_	*E*_1/2_	
CoFe‐CoFe_2_O_4_@NCs‐5/6	—	0.81	—	0.82	[129]
Co/Co_3_Fe_7_@NCNTs‐800	0.96	0.89	—	—	[130]
CoFe/Fe_3_C@CN‐900	0.996	0.865	—	—	[131]
FeCo‐Sx@NSC‐900	0.998	0.886	—	—	[132]
Fe_2_NiO_4_/FeNiS_2_	0.97	0.87	—	0.94	[133]
CoOx/Co@Co–N–C	—	0.88	—	0.83	[126]
Cu/CuO_x_‐Co_3_O_4_/NC‐700	0.91	0.81	—	—	[128]
MnF_2_/MnO_2_	0.86	0.804	0.96	0.85	[134]
Pt@Fe‐NS‐C	1.06	0.903	1.042	0.841	[135]
H‐CoTe_2_/NiTe_2_@NCBs	—	0.86	—	0.85	[136]
Co‐MnO@NC/CC	0.97	0.82	1	0.86	[127]
Co/CoS_2_/NSCNT	—	0.89	—	0.855	[137]
Gd‐2‐mim/2D‐g‐C_3_N_4_	0.85	0.6	—	—	[138]
(MOF)‐assisted CoTe_2_/MnTe_2_	0.96	0.81	0.97	0.89	[139]
MnS−BaS	—	0.838	—	0.85	[140]
CoZn/CNT@Por.NC	—	0.88	—	0.87	[141]
Ru‐NiO/Co_3_O_4_	—	0.88	0.97	0.86	[142]
Ni‐Fe/Fe_3_C@NDC	1.022	0.904	0.871	0.835	[143]
FeCo_/1:2_/PNSC	0.98	0.86	—	0.97	[144]
Au/Pd‐2 NBs/C	0.98	0.92	—	0.97	[145]
CoP/CoN@NCNRS/CC	—	0.86	—	0.8	[125]
Mo_2_C/Fe_3_C‐NC_3_	1	0.89	—	1	[146]
FePc/L‐OH/CN	1.02	0.92	1.01	0.85	[147]
CoFe@(Co_0.5_Fe_0.5_)S@NCNT/RHPC	—	0.882	—	0.864	[148]
Fe SAs‐HP	1.06	0.94	0.963	0.88	[149]
FexN@PC	0.986	0.850	—	0.834	[150]
Ni@Ni_2_P/C NSs	0.73	—	—	—	[151]
FeNSPC	0.83	0.833	—	0.836	[152]
CD/CNTs	0.95	0.81	0.98	0.83	[153]
Ni/Ni_12_P_5_@CN_x_	0.90	0.83	0.93	0.83	[154]
P‐Co_3_Fe_1_/NC‐700‐10	0.935	0.84	0.935	0.83	[155]
FeCo‐MnO@NC	1.051	0.88	—	—	[156]
Co‐NC@Nb‐TiO_x_	0.95	0.86	0.96	0.87	[157]
CeO_2_‐C/PSNC	0.95	0.78	0.96	0.87	[158]
CoMoO_4_‐RuO_2_	0.95	0.78	2	0.86	[159]
Cu NDs/Fe_2_O_3_‐NPCs	0.98	0.85	0.97	0.84	[160]
Co_3_O_4_/CeO_2_@Co/N‐CNF	0.88	0.75	—	—	[161]
Fe_2_N/CoFe_2_O_4_‐PCS	—	0.868	—	0.865	[162]
Fe,CoZn_9+9_‐NO/WC	1.041	0.889	0.974	0.863	[163]
CoNi@N‐CNT/NWs	—	0.86	—	0.84	[164]
Co/Co_9_S_8_/MnS‐NMC	0.91	0.84	0.96	0.85	[165]
Co/Co_9_S_8_‐NMC	0.92	0.83	0.95	0.88	[166]
CoVN/NC	—	0.85	—	0.86	[167]
Ag‐Cu@FeNPC	1.13	0.91	1.11	0.88	[168]

**FIGURE 11:**
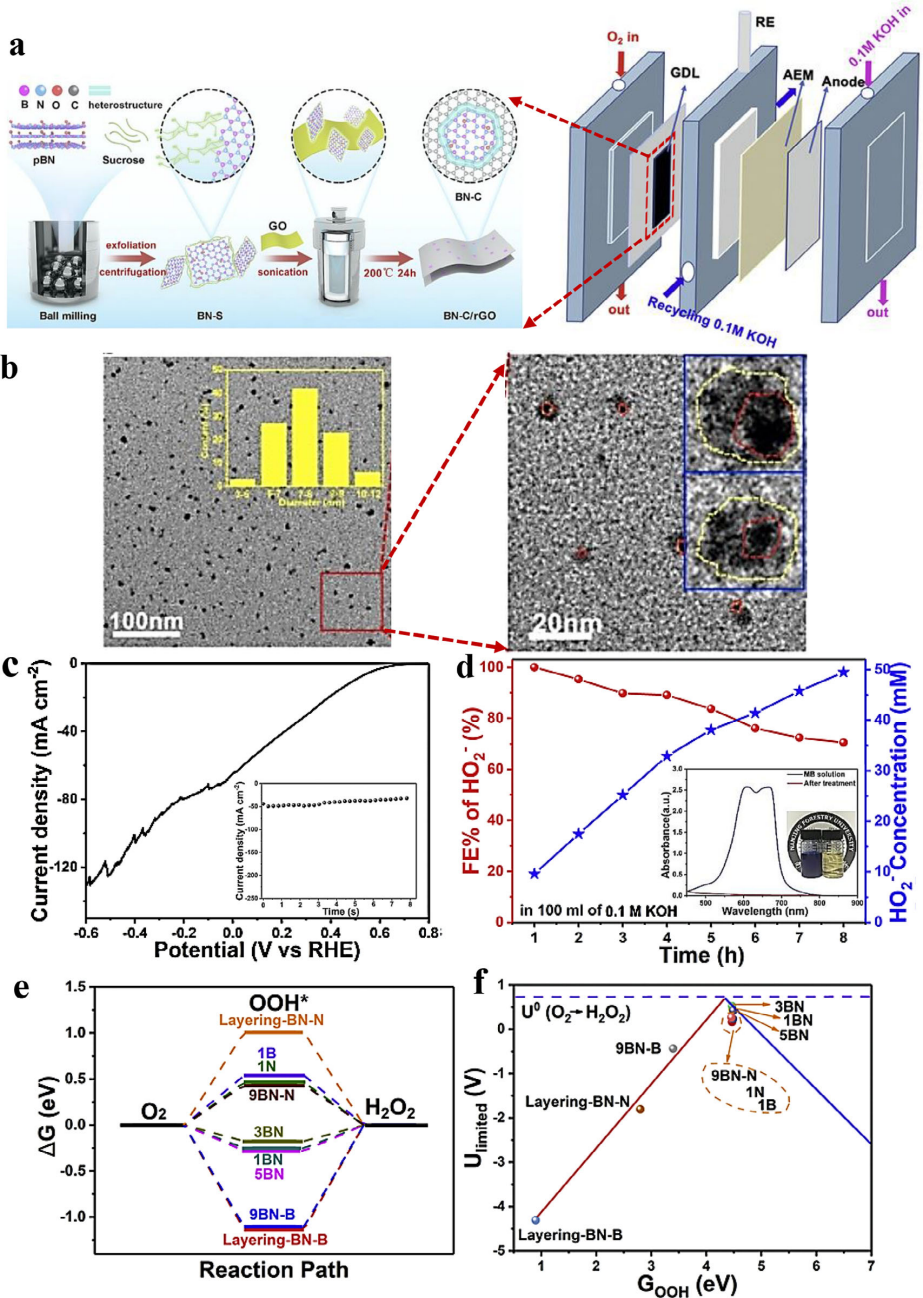
(a) Synthetic procedure for BN‐C/rGO and schematic illustration of the flow cell setup. (b) The HR‐TEM images of BN‐S dots (inset, the size distribution, the zoom‐in BN‐S dots with “core (red dotted line)‐shell (yellow dotted line)” structure). (c) Linear sweep voltammetry (LSV) curve of the flow cell with BN‐C/rGO as the catalyst at the cathode, scanned at a rate of 10 mV/s. The current‐time (i‐t) curve at 0.2 V versus RHE for hydrogen peroxide (HO^−^_2_) production in the flow cell. (d) Faradaic efficiency (FE%) of HO^−^_2_ and HO^−^_2_ concentration within the flow cell during an 8‐hour test, along with UV–vis spectra of the methylene blue (MB) solution before and after the addition of the electrolyte (inset shows photographs of the 200 ppm MB solution before and after treatment). (e) Illustration of the 2‐electron ORR reaction pathway on the optimized catalyst models. (f) Volcano plot depicting the relationship between ΔGOOH* and the 2‐electron ORR upper limit for the optimized models, with the purple dotted line indicating the equilibrium potential of O_2_/H_2_O_2_ [169]. Copyright 2023, Elsevier.

**FIGURE 12:**
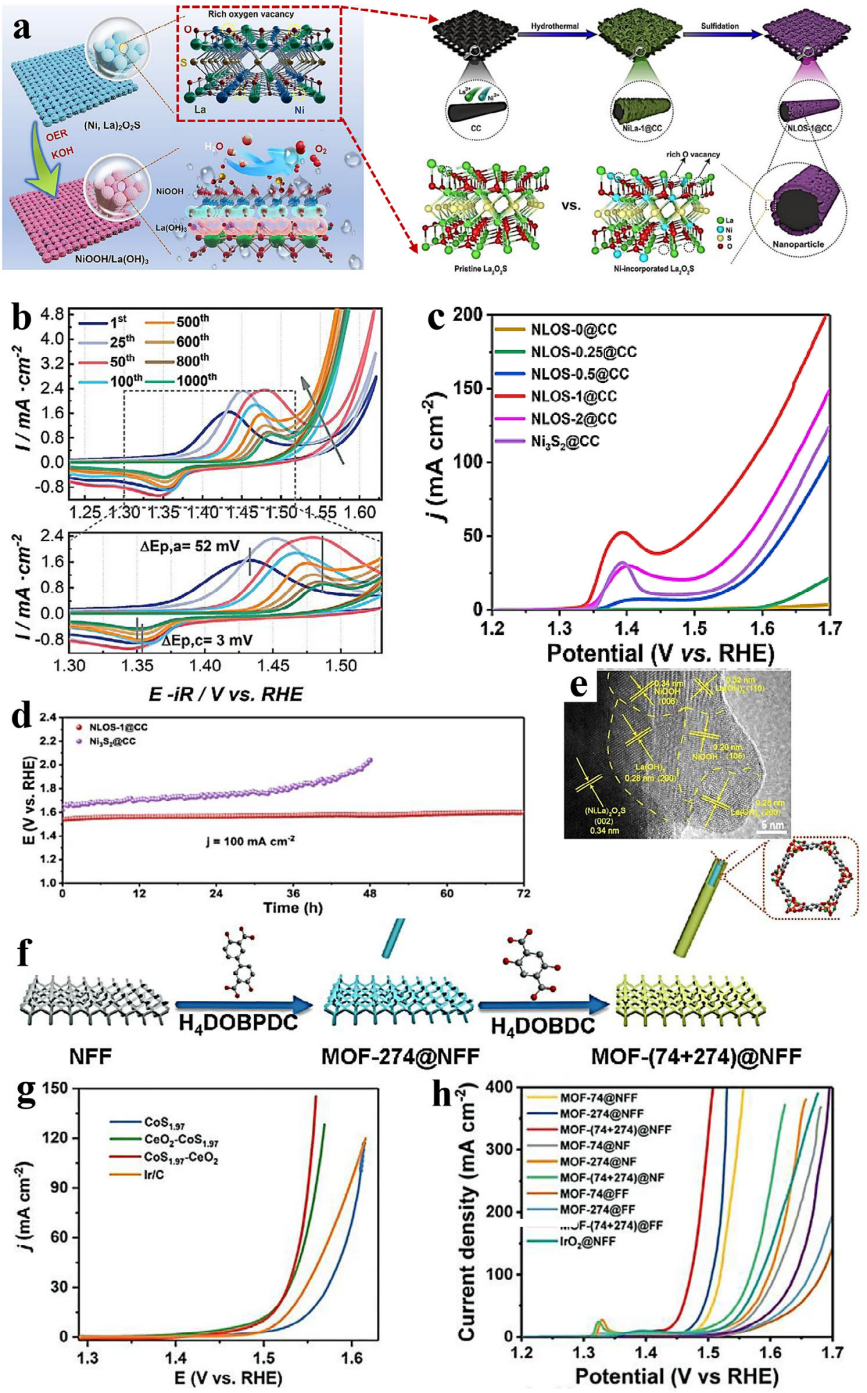
(a) Schematic illustration of the synthesis process for NLOS‐1@CC (nickel‐lanthanum oxysulfide nanoparticles on carbon cloth) [222]. (b) Cyclic voltammetry (CV) performed at a scan rate of 10 mV/s^−1^ in O_2_‐saturated 1 M KOH solution, depicting the current per geometric area over successive cycles for the LiNiO_2_ (LNO) catalyst. The shifts in anodic and cathodic peak positions (ΔEp,a and ΔEp,c) are indicated [223]. (c) IR‐corrected linear sweep voltammetry (LSV) polarization curves, showing the electrocatalytic response [222]. Copyright 2023, Springer Nature. (d) Long‐term OER CP tests of NLOS‐1@CC and Ni_3_S_2_@CC under a constant current density of 100 mA cm^−2^. (e) HRTEM image. (f) Schematic illustration depicting the synthesis of the MOF‐(74 + 274)@NFF (nickel‐iron alloy foam‐supported mixed‐metal organic framework) heterostructure [223]. (g) LSV polarization curves with 95% iR (internal resistance) correction, demonstrating the electrocatalytic performance [224]. (h) LSV curves obtained from linear sweep voltammetry measurements [223]. Copyright 2023, John Wiley and Sons.

2e^−^ ORR inter‐material heterostructures can steer selectivity by tuning reaction intermediates adsorption at the junction. Yuan et al. [169]. prepared carbon dots embedding hexagonal boron nitride/graphene (h‑BN/G) heterostructures and demonstrated their high efficiency as electrocatalysts for the 2e^−^ ORR. As illustrated in Figure 12a, sucrose‐assisted sonication and ball‐milling convert porous h‐BN (pBN) into edge‐grafted h‐BN nanodots (BN‐S) and introduce B–C/N–C covalent linkages. A subsequent hydrothermal step (200°C, 24 h) converts sucrose into graphitic carbon that grows along the h‐BN lattice, yielding carbon dots (BN‐C) with in situ‐formed h‐BN/graphite (h‐BN/G) junctions uniformly dispersed on rGO (Figure 11b). In 0.1 M KOH (RRDE), BN‐C/rGO exhibits an onset potential *E*_onset_ ≈ 0.78 V vs. RHE, 88%–95% H_2_O_2_ selectivity over 0.45–0.75 V, and ∼95% current retention after 12 h. For device‐level evaluation, a BN‐C/rGO‐coated GDL (1 mg cm^−^^2^) served as the cathode in a flow cell with a Pt anode and Ag/AgCl reference; the cell reached 100 mA cm^−^^2^ at 0.37 V vs. RHE and operated stably for 8 h at 50 mA cm^−^^2^ (Figure 11c). In alkaline media, H_2_O_2_ (reported as HO_2_^−^) production achieved 60 mmol g_cat_^−^^1^ h^−^^1^ with an initial FE > 90%, remaining > 70% after 8 h; UV–vis confirmed efficient methylene‐blue degradation (Figure 11d). DFT indicates a near‐zero Δ*G* for the rate‐determining step on a 5BN model and a planar h‐BN/G interface with ΔG_*OOH_ close to the volcano optimum (Figure 11e,f), consistent with the measured activity. The covalently coupled h‐BN/G heterojunctions tune *OOH binding and interfacial charge distribution, delivering high H_2_O_2_ selectivity and productivity in 2e^−^ ORR.

Together, these results underscore the potential of carbon‑based heterostructures as efficient, selective, and highly active sites for the 2e^−^ ORR. Looking ahead, continued progress will hinge on rational catalyst design, synthetic innovation, systematic heterojunction heterostructure constructing (tuning work‑function/band alignment, interfacial dipoles, heteroatom/defect distributions, wettability, and O_2_/H_2_O_2_ transport), alongside device‑level evaluations (e.g., flow‑cell performance) and environmental remediation tests to guide practical deployment.

Oxygen evolution reaction (OER)—the anodic half‑reaction of water electrolysis—is central to solar‑fuel production and electrochemical energy‑storage technologies. Although iridium‑ and ruthenium‑based materials set the activity benchmark, their scarcity and cost motivate the search for economical, efficient alternatives [170]. OER generally proceeds through four proton‑coupled electron‑transfer (PCET) steps that include water adsorption/activation and, ultimately, dioxygen formation. Along this pathway, the catalyst stabilizes key oxygen‑containing intermediates—*OH, *O, and *OOH. OER kinetics are typically sluggish because of the reaction's complex multi‑electron, multi‑step nature. Accordingly, catalyst design must balance the adsorption strengths of intermediates—consistent with the Sabatier principle—so that binding is neither too strong nor too weak; this enhances the reaction rate and reduces energy input. OER is often discussed in terms of two limiting pathways [31, 32]: an associative route in which O─O bond formation via OOH precedes the final PCET steps, stabilizing the peroxide intermediate; and a dissociative route in which O─O coupling occurs after PCET on more reactive O‑type species. Well‑engineered heterointerfaces provide strong electronic coupling that tunes charge transport and intermediate adsorption, thereby boosting OER activity. Precise control of interfacial structure allows simultaneous modulation of electronic properties and chemical stability—both critical to performance. As with the ORR, the OER pathway depends on catalyst composition, surface coverage, and operating conditions. Despite extensive study, achieving a high activity together with long‑term stability remains challenging, warranting further mechanistic insight and materials optimization. Notably, the tunability of heterostructures torward the adsorption energies of O‑ and H‑containing intermediates holds promise for OWS. Table 2 summarizes representative works on OER that employed heterostructured catalysts.

**TABLE 2:** Composition, electrolyte conditions, and OER performance of heterostructured electrocatalysts. Composition and OER performance of heterostructured electrocatalysts. Tafel slopes and overpotentials were obtained at 10 mA cm^−^^2^. Unless otherwise specified, all measurements were conducted in 1 M KOH.

Electrocatalyst	Tafel slope [mV dec^−1^]	Overpotential [mV]	Ref.
Mo_15_S_19_/Ni:FeOOH	93.7	214	[171]
CoNi_5_S_8_‐Ni_2_P‐FeP_2_	34	215	[172]
NiCu/FeNi_2_S_4_	49	306	[173]
CoP@NiFe LDH^a^	44.4	260	[174]
NiFe‐LDH@Co_9_S_8_‐Ni_3_S_2_/NF^a^	45.74	298	[175]
CoS_2_@(Fe,Ni)S_2_	54	—	[176]
Fe_2_NiO_4_/FeNiS_2_	45.28	296	[133]
Co_3_Mo/Mo_2_C@NC	59.3	282	[177]
Vo‐NiO/NiFe_2_O_4_	39.4	261	[178]
Fe_2_P/NiCoP n‐n^a^	76.3	291	[179]
CoSe/CoO	38.36	—	[180]
NiFeP/CoP	70	274	[181]
FeCoNi‐LDH@CF	33.35	207	[182]
FeNiP‐NiMoOx@CoNiS	39.9	229.8	[183]
FeP_4_/CoP/C	41.0	258	[184]
Cu‐Co^b^	—	346	[128]
NiFeLDH/RuSe_2_	69	268	[185]
CoCr‐LDH/NiO/NF	70.92	253	[186]
NiS/NiMo	87	245	[187]
Co_3_S_4_@NiFe‐LDH	38	235	[188]
Mo‐NiO@NiFe‐LDH	30.4	—	[189]
Fe−Ni−CoOOH‐TP*A*	39	236	[190]
NiCo_2_O_4_/NiO	66.7	288	[191]
NiFe LDH/MOF	32.5	196	[192]
MoS_2_/NiFeCr LDH	61	224	[193]
CoTe@FeOOH	69.9	—	[194]
Ni_3_S_2_–FeMoO_4_	75.77	262	[195]
CoFeB/NiPS_3_	96	—	[196]
FeOOH/Ni_3_S_2_	73	—	[197]
CuCo_2_O_4_/NiCo_2_O_4_ (CCO/NCO)	93	>250	[198]
RuO_2_−CeO_2_ ^c^	58.9	180	[199]
NiFeCo─OH/NiTe	105	—	[200]
Co‐MnO@NC/CC	64.5	—	[127]
MoS_2_/NiS_2_/Ni_3_S_4_	117	210	[201]
I‐Ni(OH)_2_/Co(OH)_2_@NF	—	276	[202]
NiFe‐MOF‐74/NiFe‐LDH	39.8	159.7	[203]
CoP@CoOOH/CP	131.8	200	[204]
CoNiLDH/FeOOH n–n	60	250	[205]
a‐CoS/Ni_3_S_2_	53	192	[206]
NiFe‐LDH/Co_3_O_4_ p‐n	81	274	[207]
Fe_2_O_3_/ZnCo_2_O_4_	71.8	261	[208]
Ni_0.8_5Se‐O/CN	82.5	240	[209]
Ni_3_S_2_/NiO (NiSO‐2) p‐n	46	240	[210]
t‐BTO@NiFe‐LDH	38.3	186	[211]
(FeOOH/CoP/3D NF	32	234	[212]
NiFe‐LDH/SnS	53.6	310	[213]
Co@Ni/Fe‐MS/MOF	52.37	229	[214]
NiFe‐LDH@CoP‐Ni_5_P_4_	38.4	179	[215]
CoB_2_O_4_@FeOOH/NF	116.9	255	[216]
Ni_2_P–MoP@NC	53	249	[217]
Ru/d‐NiFe LDH	60.48	220	[218]
FeCoNi LDH/CuO/Cu	63.8	—	[219]
CoP/CoN@NCNRs/CC	60.2	—	[125]
Mo_5_N_6_‐Ni_3_S_2_ HNPs/NF	42	190	[220]
NiFe_0_․_3_ LDH	50.7	230	[221]

^a^ Measured in 1 M KOH + 0.5 M NaCl.

^b^ Measured in 0.1 M KOH.

^c^ Measured in 0.5 M H_2_SO_4_.

In alkaline OER, a La–Ni oxysulfide on carbon cloth (NLOS@CC) delivers an overpotential of 260 mV at 50 mA cm^−^^2^ and maintains 100 mA cm^−^^2^ for 72 h, outperforming typical Ni‐based catalysts. The material is derived from a La_2_O_2_S prototype: a highly crystalline Ni/La hydroxide nanosheet array (NiLa‐1@CC) is grown on carbon cloth and then sulfurized to form oxygen‐vacancy‐rich (Ni,La)_2_O_2_S nanoparticles (NLOS‐1@CC) (Figure 12a). Figure 12c identifies NLOS‐1@CC as the best‐performing composition in the series. The authors attribute the activity to a heterostructure that maximizes active‐site exposure, accelerates charge transfer, preserves structural integrity, and lowers the adsorption free‐energy barrier along the OER pathway. Post‐reaction HRTEM reveals NiOOH and La(OH)_3_ lattice fringes in close contact with residual (Ni,La)_2_O_2_S, confirming an in situ reconstructed multiphase heterointerface under OER conditions (Figure 12e). This heterostructure likely increases accessible active sites and promotes interfacial charge transfer, consistent with the sustained current during long‐term electrolysis (Figure 12d) [222]. La(OH)_3_/NiOOH composite. A distinct composite comprising lanthanum hydroxide [La(OH)_3_] and nickel oxy(hydroxide) (NiOOH) was also prepared. CV data (Figure 12b) reveal deprotonation‐driven changes in LiNiO_2_ during cycling that correlate with enhanced activity. Together with complementary measurements, the results indicate in situ formation of a self‐adaptive LiNiO_2_/NiOOH heterostructure, which underpins the high OER performance under alkaline conditions [223]. Bimetallic MOF mixtures have likewise been explored for OER. A representative example is the MOF‑74/MOF‑274 heterostructure grown in situ on nickel–iron alloy foam (NFF) via a two‑step self‑assembly process, yielding MOF‑(74+274)@NFF (Figure 12f). As shown in Figure 12h, MOFs@NFF exhibits the highest OER activity, followed by MOFs@NF and MOFs@FF. These results underscore the role of partial Fe→Ni electron transfer in enhancing OER performance. Consistent with experimental observations and theoretical calculations, the MOF‑(74+274)@NFF heterojunction finely tunes the electronic structure of the active sites and strengthens the adsorption of oxygen‑containing intermediates, thereby optimizing the Gibbs free energy landscape and lowering the rate‑determining‑step barrier to boost activity. Two self‐supported CeO_2_/CoS_1_․_97_ heterostructures with distinct spatial architectures were constructed. Figure 12g shows that pre‐oxidation with either CeO_2_ or CoS_1_․_97_ decorations triggers earlier redox transitions, with CeO_2_–CoS_1_․_97_ exhibiting the lowest transition potential. By tailoring the CeO_2_ functional layer, these heterostructures regulate the interfacial electronic structure, stabilize the interfacial perimeter, and promote operando reconstruction, jointly enhancing OER activity [224]. Taken together, these studies demonstrate that rational heterostructure design can maximize active‑site exposure, modulate the electronic structure, and tailor intermediate adsorption, culminating in substantial improvements in OER performance.

Beyond freshwater alkaline electrolysis, direct seawater or saline‐water electrolysis is attracting growing attention for coastal or offshore hydrogen production. In chloride‐containing media, the anodic reaction must deliver high OER activity while suppressing the competing chlorine evolution reaction, and the electrode must tolerate chloride‐induced corrosion and hypochlorite attack. These coupled requirements make activity‐only benchmarking insufficient [225]. Recent studies indicate that heterostructure/interface engineering can help reconcile activity, selectivity, and durability by tailoring local coordination chemistry and interfacial charge redistribution to disfavor Cl^−^ adsorption while stabilizing oxygenated intermediates. For example, Ren et al. reported a hierarchical NiPOx@NiFe layered double hydroxide nanoarray on Ni foam that reaches 1000 mA cm^−^^2^ at an overpotential of 352 mV and sustains seawater oxidation for 600 h in alkaline seawater, highlighting the benefits of phosphate‐derived interphases for chloride tolerance [226]. Complementarily, electrolyte‐level interfacial regulation has also proven effective: hexafluorophosphate (PF_6_^−^) additives can accumulate at the electrode surface and within NiFe LDH interlayers to block Cl^−^ and stabilize the active phase, enabling ampere‐level seawater oxidation over thousands of hours [227]. These seawater‐oriented design rules are consistent with broader interface‐driven electronic‐structure regulation concepts established for OER under harsh conditions [228].

**FIGURE 13:**
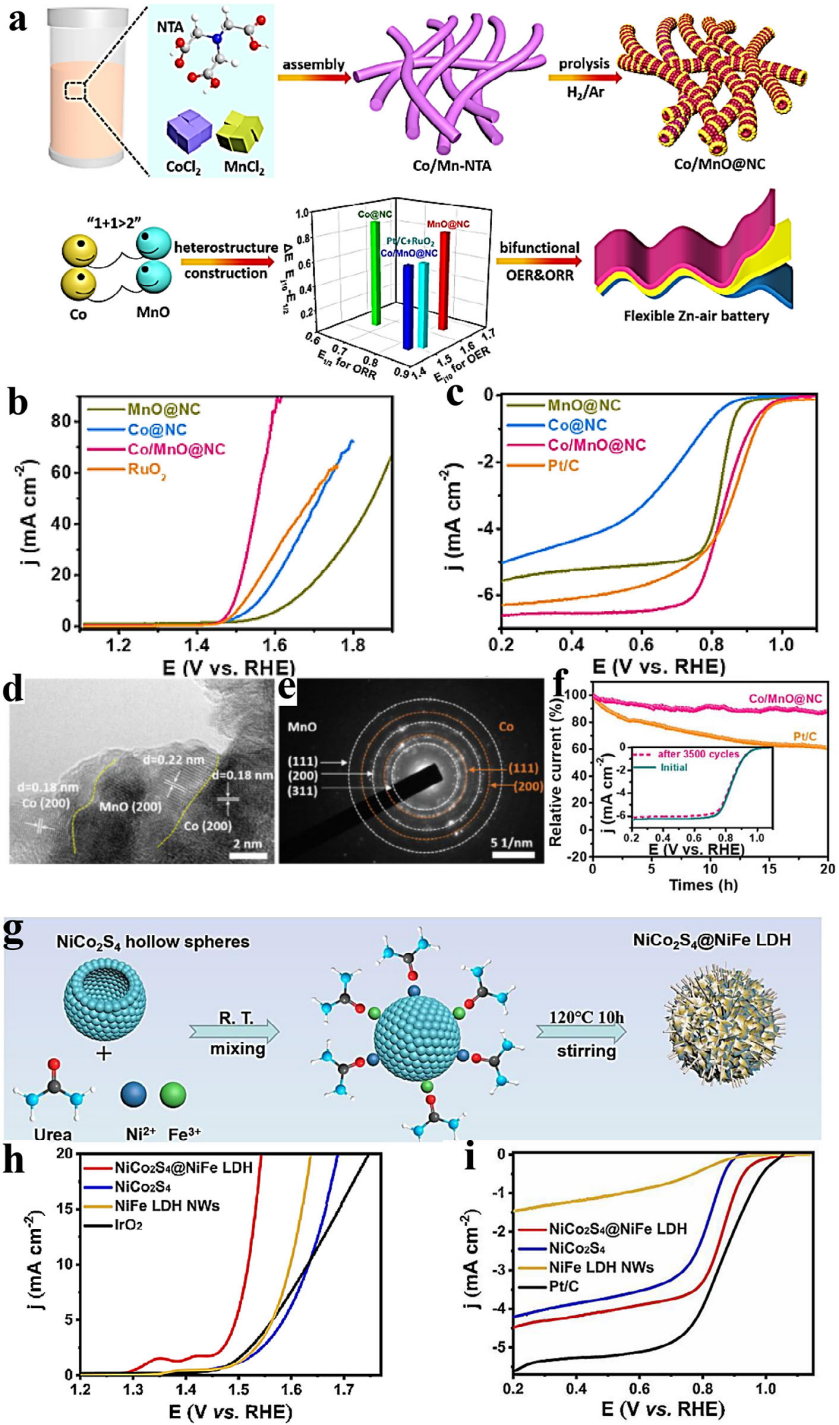
(a) Schematic depiction of the synthetic pathway for Co/MnO@NC. (b) OER polarization curves of the prepared catalysts and RuO_2_ in 1 M KOH. (c) ORR polarization curves of different catalysts and Pt/C in 0.1 M KOH at 1600 rpm [229]. Copyright 2021, Elsevier. (d,e) HRTEM and SAED analyses of Co/MnO@NC reveal a well‐defined Co–MnO heterointerface with clear lattice fringes and confirm the coexistence of crystalline Co and MnO phases. (f) ORR chronoamperometric response of Co/MnO@NC and Pt/C at a constant potential of 0.7 V (inset: ORR activities of Co/MnO@NC before and after 3500 cycles). (g) Schematic representation of NiCo_2_S_4_@NiFe LDH. (h) OER polarization curves of NiCo_2_S_4_@NiFe LDH, NiCo_2_S_4_, NiFe LDH NWs, and IrO_2_. (i) ORR polarization curves of NiCo_2_S_4_@NiFe LDH, NiCo2S4, NiFe LDH NWs, and Pt/C at 1600 rpm in 0.1 M KOH [230]. Copyright 2021, Elsevier.

The above discussion examines how intra‐phase and heterointerfaces impact ORR/OER performance in both single‐phase and multicomponent catalysts. However, single‑component catalysts are often limited by the density of accessible active sites, making it challenging to achieve high efficiency for both reactions simultaneously. To address this limitation, researchers are developing bifunctional electrocatalysts that materials that catalyze two different reactions—now a central theme in high‑performance catalyst design. Niu et al. [229]. reported a bifunctional Co/MnO@NC catalyst, first synthesizing one‑dimensional (1D) Co/Mn–nitrilotriacetic acid(NTA) coordination‑polymer nanowires via a solvothermal route. During this process, the organic chelator NTA coordinates with Mn^2^^+^ and Co^2^^+^ via its carboxylate groups, directing chain growth to form 1D Co/Mn–NTA coordination polymers (Figure 13a). As shown in Figure 13b, Co/MnO@NC exhibits notable OER activity, with an onset overpotential of 235 mV and an overpotential of 260 mV at 10 mA cm^−^^2^. Beyond OER, Co/MnO@NC also delivers competitive ORR performance: the LSV curve in Figure 13c indicates *E*_onset_ = 0.96 V and *E*_1/2_ = 0.83 V, approaching commercial Pt/C (*E*_onset_ = 0.98 V, *E*_1/2_ = 0.85 V). HRTEM resolves distinct lattice fringes attributable to Co and MnO that converge at a well‐defined interface (Figure 13d), while the corresponding SAED pattern confirms the coexistence of crystalline Co and MnO phases (Figure 13e). Consistent with this interface‐engineered architecture, Co/MnO@NC shows enhanced durability compared with Pt/C during prolonged operation and after extensive cycling (Figure 13f). Together, these experimental results underscore the pivotal role of Co/MnO heterostructure constructing in enabling high electrocatalytic activity. Notably, the strategy extends to Fe/MnO@NC and Ni/MnO@NC analogues, which also show excellent bifunctional ORR/OER performance.

**FIGURE 14:**
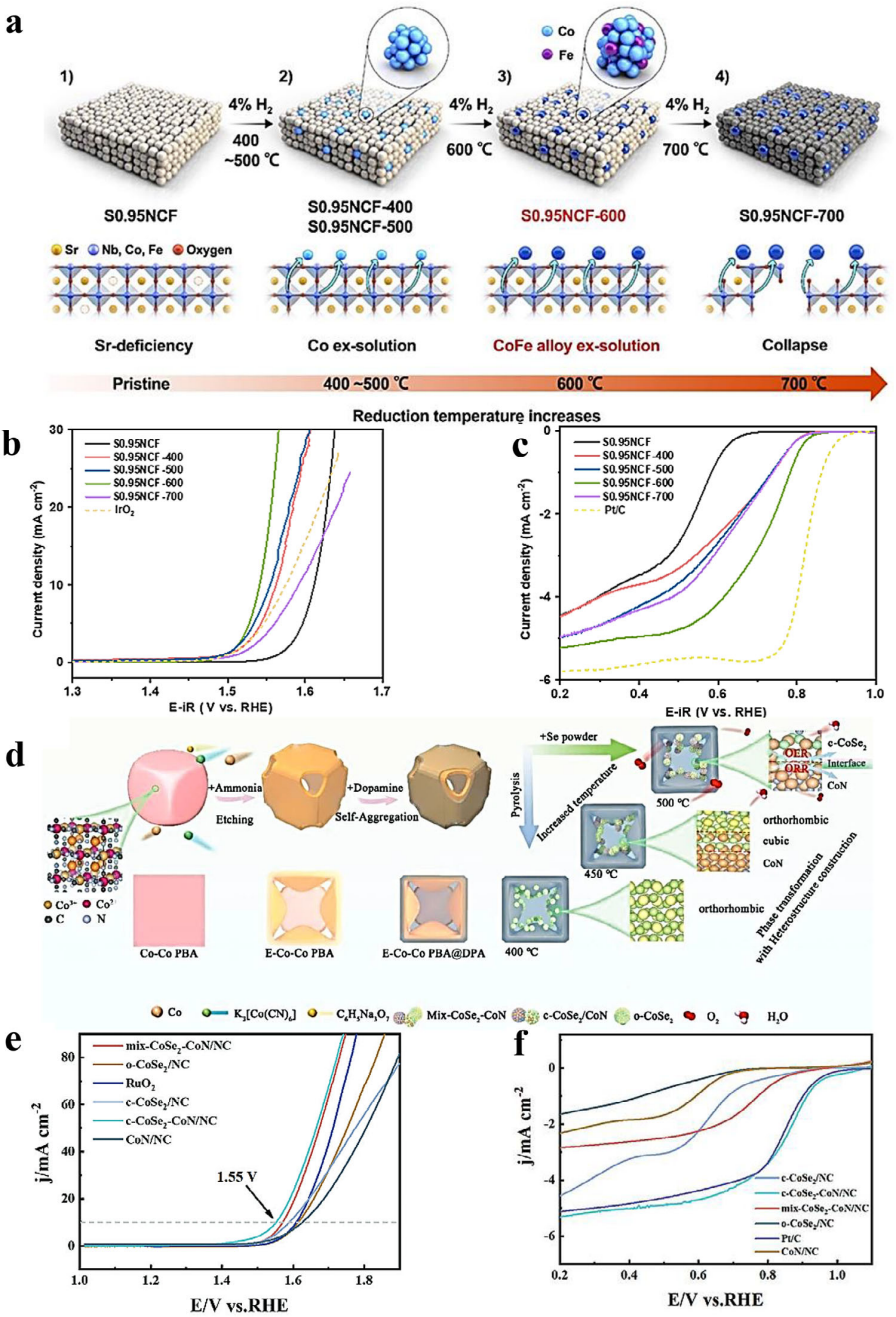
(a) Schematic representation of the synthetic process via ex‐solution method. (b) OER LSV polarization curves for S0.95NCF and S_0.95_NCF‐X in O_2_‐saturated 0.1 M KOH electrolyte. (c) ORR LSV polarization curves for S_0.95_NCF and S_0.95_NCF‐X in O_2_‐saturated 0.1 M KOH electrolyte with continuous electrode rotation at 1600 rpm [231]. Copyright 2022, Elsevier. (d) Synthesis pathway for o‐CoSe_2_/NC, Mix‐CoSe_2_‐CoN/NC, and c‐CoSe_2_‐CoN/NC catalysts. (e) LSV curves (2 mV s^−1^ and 1600 rpm). (f) LSV curves at 5 mV s^−1^ and 1600 rpm [232]. Copyright 2023, John Wiley and Sons.

In a related solvothermal approach (Figure 13g), NiCo_2_S_4_ hollow spheres served as a hydrophilic nucleation scaffold that uniformly adsorbed Fe^3^^+^ and Ni^2^^+^, promoting even surface coverage [230]. Subsequent in situ hydrolysis yielded NiFe‑LDH nanowires and nanosheets, forming the bifunctional NiCo_2_S_4_@NiFe‑LDH catalyst. For OER, as shown in Figure 13h, NiCo_2_S_4_@NiFe‑LDH achieves 10 mA cm^−^^2^ at an overpotential of only 287 mV. Relative to NiCo_2_S_4_ (*E*_onset_ ≈ 0.89 V, *E*_1/2_ ≈ 0.80 V, *j*_L_ ≈ 4.21 mA cm^−^^2^) and NiFe‑LDH nanowires (*E*_onset_ ≈ 0.85 V, *E*_1/2_ ≈ 0.67 V, *j*_L_ ≈ 1.47 mA cm^−^^2^), NiCo_2_S_4_@NiFe‑LDH shows *E*_onset_ = 0.97 V (at 0.2 mA cm^−^^2^), *E*_1/2_ = 0.85 V, and *j*_L_ = 4.49 mA cm^−^^2^. Compared with NiFe‐LDH nanowires, NiCo_2_S_4_ contributes the ORR‐active and highly conductive scaffold, while NiFe–LDH supplies the OER‐active sites; the NiCo_2_S_4_/NiFe‐LDH heterointerface couples these complementary roles to deliver the observed bifunctionality (Figure 13h,i). This highlights a general design principle for non‐precious heterostructures: combine an ORR‐active, conductive backbone with an OER‐active LDH overlayer to achieve efficient, durable bifunctional electrocatalysis. Another study prepared SrNb_0_._1_Co_0_._7_Fe_0_._2_O_3_(SNCF) and Sr_0_._95_Nb_0_._1_Co_0_._7_Fe_0_._2_O_3_–δ(S_0.95_NCF) perovskite oxides via a solid‑state reaction followed by high‑energy ball milling (Figure 14a). Surface‐regulated S_0.95_NCF‐X—S_0.95_NCF reduced at X °C for 1 h in 4% H_2_/Ar—outperforms the unregulated sample in both ORR and OER: it shows more positive ORR *E*_onset_/*E*_1/2_ and higher *j*_L_, and reaches a given OER current density at lower potentials (Figure 14b,c). These findings offer practical guidance for designing heterogeneous electrocatalysts for multifunctional energy devices [231]. Xu et al. [232]. prepared cobalt–cobalt Prussian blue analog (Co–Co‐PBA) nanocubes by liquid‐phase coprecipitation. Open‐hollow “E‐Co–Co‐PBA” nanocages were then obtained by site‐selective chemical etching along the cubes’ body diagonals, which preserves the external cubic morphology while creating corner apertures and an internal cavity. Dopamine (DPA) was in situ polymerized on the nanocage surface to form E‐Co–Co‐PBA@DPA, providing a robust sheath that maintains the cubic architecture during subsequent vapor‐deposition/annealing steps. Finally, using elemental Se as the source, metastable o‑CoSe_2_ was converted to phase‑stable c‑CoSe_2_ under N_2_ (Figure 14d). The resulting heterojunction exhibits strong interfacial adhesion and lower interfacial charge‐transfer resistance, and its band alignment/charge redistribution improves the adsorption energetics of OER intermediates, thereby boosting activity (Figure 14e). Additionally, relative to o‑CoSe_2_/NC and mix‑CoSe_2_–CoN/NC, the c‑CoSe_2_–CoN/NC composite exhibits substantially enhanced ORR activity, indicating that interface/phase‑transformation is critical to promoting the ORR activity of CoSe_2_. In particular, strong c‑CoSe_2_–CoN coupling facilitates interfacial electron transfer between the catalytic surface and intermediates, thereby accelerating ORR (Figure 14f). This study illustrates that heterostructure constructing offers a targeted route to tune the bifunctional activity of phase‑engineered metal selenides. The reported synthetic and interfacial strategies are broadly generalizable across catalyst families and open avenues for designing efficient energy‑conversion materials. Future work should prioritize standardized bifunctionality metrics, operando tracking of interface reconstruction, and constant‐potential, explicitly solvated DFT and microkinetic modeling to close the loop between structure, mechanism, and device performance.

### 4.2 Hydrogen Oxidation Reaction (HOR)

HOR proceeds via adsorption of H_2_ on the catalyst surface, dissociation of the H–H bond, formation of adsorbed hydrogen (H_ad_), and subsequent electro‑oxidation to generate protons (acidic media) or water (alkaline media) [34]. HOR is pivotal to both anion exchange membrane fuel cells (AEMFCs) and proton exchange membrane fuel cells (PEMFCs). It is usually facile (non‐RDS) in PEMFCs on PGM catalysts but often rate‐determining in alkaline AEMFCs. AEMFCs do permit non‐platinum‐group metal(non‐PGM) catalysts mainly for ORR; by contrast, alkaline HOR still predominantly uses PGM catalysts today. Nevertheless, Pt exhibits markedly lower HOR activity in alkaline AEMFC environments than in PEMFCs, often requiring higher Pt loadings and motivating the search for cost‑effective PGM alternatives. While HOR and HER both involve the adsorbed‐hydrogen intermediate H_ad_, their activity trends can diverge, so HER‐active metals are not automatically HOR‐active. In practice, HOR activity still predominantly arises from PGMs (notably Pt), consistent with differences in hydrogen/‐OH adsorption and surface oxidation under operating conditions [233, 234]. To reduce PGM reliance and enhance economic viability, recent efforts have focused on non‑noble‑metal and PGM‑lean catalysts, with particular emphasis on alkaline HOR where earth‑abundant elements are attractive substitutes for Pt‑based materials [235]. Heterostructure engineering offers a powerful lever to tune the binding energies of H_ad_ and OH_ad_, modulate interfacial charge distribution, and create complementary active sites, thereby boosting HOR activity under alkaline conditions. Among PGMs, ruthenium is substantially less expensive than Pt and provides a favorable balance of oxygen affinity and hydrogen binding energy, enabling optimal adsorption of OH_ad_ and H_ad_ at low anodic potentials—attributes essential for rapid alkaline HOR kinetics. A deeper mechanistic understanding of how heterostructures operate—and how to exploit interfacial design to optimize reaction kinetics—remains central to developing efficient, low‑cost HOR catalysts. Accordingly, Table 3 compiles representative hetero‐structured catalysts reported in recent years. Notably, several of these systems achieve mass activities comparable to—or even exceeding—those of conventional Pt on carbon (Pt/C), reinforcing that judiciously engineered heterointerfaces can furnish additional, highly active sites that enhance reactant adsorption/activation, expand catalyst–reactant contact, and ultimately improve catalytic efficiency.

**TABLE 3:** Basic information and HOR performance of heterostructured electrocatalysts. Mass activities are reported at the overpotentials listed in the table. Unless otherwise specified, all measurements were performed in 0.1 M KOH.

Electrocatalyst	Mass activity at 50 mV overpotential (A g^−1^)	Pt/C	Overpotential (mV)	Reference
Ru‐RuO_2_/C 250NA	618.37 A gRu^−1^	366.01 A gPt^−1^	50	[236]
Ru/WO_2.9_/C	8.29×10^−3^ A mg_NM_^−1^	0.38×10^−3^ A mgNM^−1^	50	[237]
Ru‐RuS_2_@C	2.13 × 10^3^ A gRu^−1^	—	50	[238]
Ni_17_W_3_/WO_2_/rGO	2.427× 10^−2^ A g^−1^ Ni	—	50	[239]
MoP–Mo_2_C/C	—	—	—	[240]
Ni/V_2_O_3_	42.1 A gNi^−1^	—	50	[241]
Ni/Ni_0.2_Mo_0.8_N/rGO	2.9 mA cm^−2^	2.3 mA cm^−2^	100	[242]
Ru–RuO_2_/C	2.5 × 10^3^ A g^−1^	0.42× 10^3^ A g^−1^	50	[243]
RuO_2_–PdO NWs/C	1061 A gRuPd^−1^	338 A gP^t‐1^	—	[244]
a‐Pt_53_Ru_47_ NWs/C	1.37× 10^4^ A gPGM^−1^	∼0.21× 10^4^ A gPGM^−1^	25	[245]
CoO/CoMoO_4_ NF	—	—	—	[246]
Ni–MoO_2_/NF^a^	3.26 mA cm^−2^	4.12 mA cm^−2^	50	[247]
PdRh_0_._05_/C	1.16 A gPGM^−^^1^	0.24 A gPGM^−1^	50	[248]

^a^ Measured in 1 M KOH.

**FIGURE 15:**
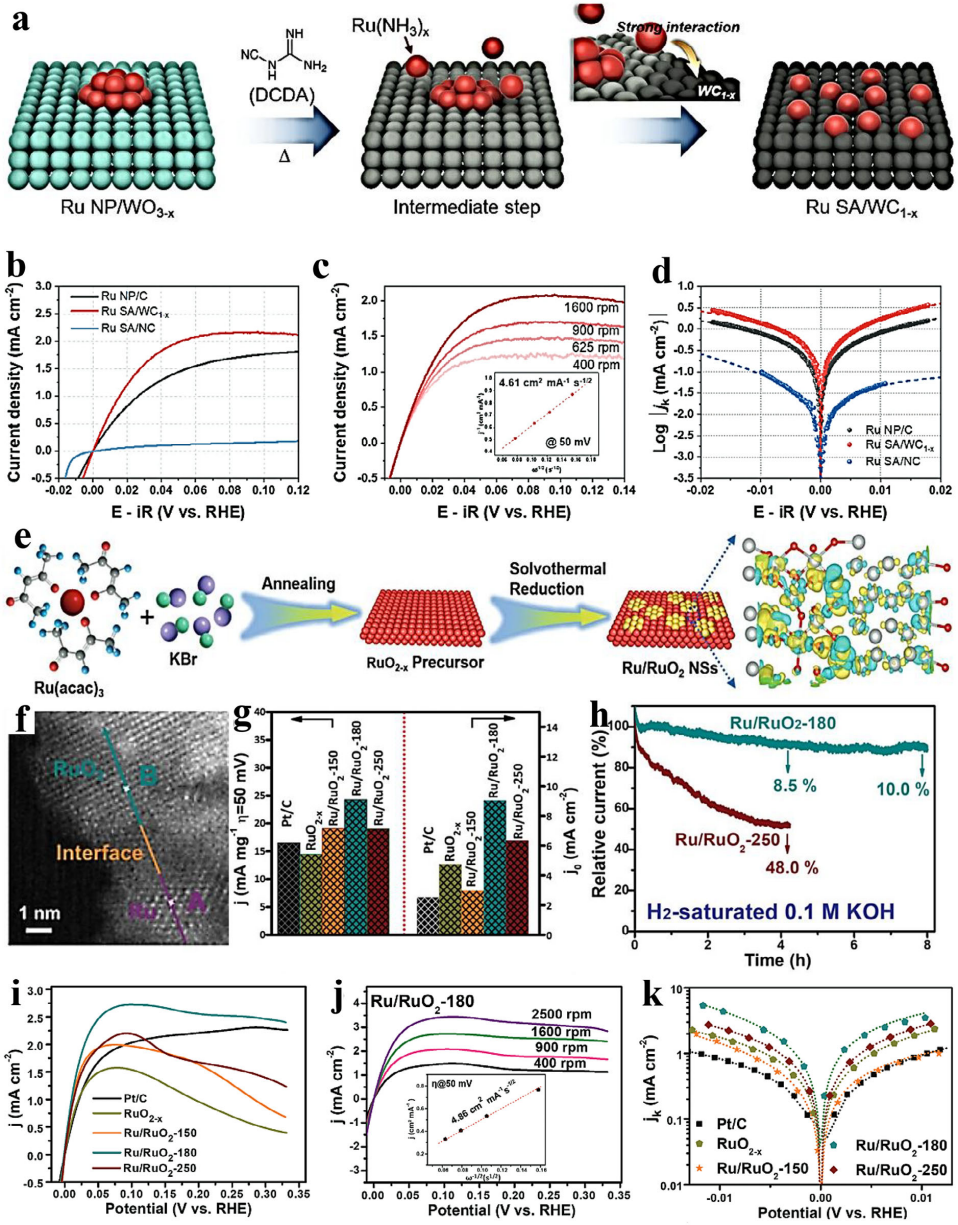
(a) Schematic representation of the synthesis process for Ru SA/WC_1−x_. (b) HOR polarization curves of Ru NP/C, Ru SA/WC_1−x_, and Ru SA/NC in H_2_‐saturated 0.1 M KOH with a scan rate of 1 mV s^−1^ at 1600 rpm. (c) HOR polarization curves at various rotating speeds of Ru SA/WC_1−x_. Inset: Koutecký–Levich plot at an overpotential of 50 mV [249] Copyright 2023, John Wiley and Sons. (d) Tafel plots with Butler–Volmer fitting solid lines. (e) Schematic illustration of the synthesis of Ru/RuO2. (f) HAADF‐STEM image. (g) Comparison of *j*_0_ and mass‐normalized current density at an overpotential of 50 mV of different catalysts. (h) Relative chronoamperometry response of Ru/RuO_2_‐180 and Ru/RuO_2_‐250 in H_2_‐saturated 0.1 M KOH solution at 0.1 V (vs. RHE). (i) Geometric area‐normalized LSV curves of different catalysts. (j) LSV curves of Ru/RuO_2_‐180 at various rotation rates, with the inset showing the Koutecký–Levich plot at an overpotential of 50 mV. (k) Linear fitting based on the Butler–Volmer equation of the kinetic current density at 1600 rpm. (Symbols represent calculated j_k_ based on experimental data, and dashed lines indicate fitting results) [250] Copyright 2023, John Wiley and Sons.

Park et al. [249] designed ruthenium single‑atom catalysts (Ru SACs) anchored on tungsten carbide (Ru SA/WC_1__−__x_; Figure  15a). At 0.02 V overpotential, it outperforms Ru NP/C (0.83 mA cm^−^^2^) and commercial Pt/C (0.95 mA cm^−^^2^; Figure 15b). With increasing rotation rate in RDE test, the current density rises as expected from enhanced mass transport; the Koutecký–Levich slopes for Ru SA/WC_1__−__x_ and Ru NP/C are 4.61 and 4.58 cm^2^ mA^−^^1^ s^−^^1^/^2^, respectively—close to the theoretical 4.87 cm^2^ mA^−^^1^ s^−^^1^/^2^ for a two‑electron HOR (Figure 15c). To probe intrinsic activity, the *j*_0_ was extracted from kinetic current (*j*_k_) obtained via Koutecký–Levich analysis, and the results indicate a superior noble‑metal utilization in Ru SA/WC_1__−__x_ compared to Ru SA/NC and Ru NP/C (Figure  15d). This study also compares Pt single‑atom catalysts in acidic vs. alkaline media and shows how heterostructure constructing improves the durability of Ru‑based HOR catalysts. In a complementary study, RuO_2__−__x_ nanosheets were prepared by heating Ru(acac)_3_ with KBr in air, followed by solvothermal reduction in ethylene glycol to afford Ru/RuO_2_ heterostructured nanosheets with tunable Ru^0^/(Ru^4^^+^ + Ru^0^) ratios (Figure 15e) [250]. The in‐plane heterostructure reduced at 180°C (Ru/RuO_2_‐180) shows the highest HOR current density over the tested potential range, whereas RuO_2__−__x_ is the least active (Figure 15i). Compared with Ru/RuO_2_‐250, Ru/RuO_2_‐180—rich in Ru–RuO_2_ interfaces—suppresses Ru oxidation within 0–0.33 V vs. RHE, avoiding the ∼0.1 V current loss and improving HOR durability (Figure 15e). High‐resolution imaging directly evidences the in‐plane Ru–RuO_2_ heterointerface, where Ru and RuO_2_ lattice domains coexist across a well‐defined boundary (Figure 15f). Activity benchmarking further shows that Ru/RuO_2_‐180 achieves the highest HOR performance: its exchange current density and mass‐normalized current (evaluated at 50 mV) outperform the other compositions, consistent with an optimized balance between metallic Ru and oxide domains (Figure 15g). In addition, Ru/RuO_2_‐180 exhibits markedly improved durability compared with Ru/RuO_2_‐250 under potentiostatic operation, retaining most of its initial current while Ru/RuO_2_‐250 suffers rapid decay, indicative of mitigated Ru oxidation enabled by the abundant Ru–RuO_2_ interfaces (Figure 15h). RDE polarization follows Koutecký–Levich behavior (Figure 15j); comparisons therefore use the *j*_k_. Butler–Volmer analysis further confirms markedly accelerated HOR kinetics for Ru/RuO_2_‐180 (Figure 15k). Consistent with experiment and DFT, adjacent Ru and RuO_2_ domains provide complementary optimal adsorption sites for H and OH, respectively, making the Ru(100)/RuO_2_(200) interface a preferred region for rapid alkaline HOR.

Overall, although HOR in AEMFCs is promising, achieving high rates in alkaline media without heavy PGM loadings remains challenging. Heterostructure and single‑atom strategies—exemplified by Ru/RuO_2_ and Ru SA/WC_1__−__x_—tune H/OH adsorption energetics, create abundant interfacial sites, and improve metal utilization and stability. These advances deepen mechanistic understanding and open pathways to low‑cost, high‑performance anodes for AEMFCs.

### 4.3 Hydrogen Evolution Reaction (HER)

Water electrolysis offers a clean route to high‑purity hydrogen and a sustainable alternative to fossil fuels. In this process, hydrogen evolution reaction is a key half‑reaction governed by the Volmer, Heyrovsky, and Tafel steps [251, 252]. In alkaline electrolytes, the Volmer step proceeds via water adsorption and dissociation to generate Had, whereas in acid it involves the electrochemical combination of H^+^ and electrons to form H_ad_ [253, 254]. Hydrogen is then produced either via the Heyrovsky pathway—electrochemical desorption of H* with water in alkaline media or with protons in acid—or via the Tafel pathway, in which two adsorbed hydrogen species recombine to form H_2_. Across these pathways, adsorbed hydrogen is the sole reaction intermediate; accordingly, the reaction rate is dictated by its adsorption energy. The design of high‑performance HER electrocatalysts seeks to minimize overpotential, accelerate kinetics, increase energy efficiency, and ensure durability. According to the Sabatier principle, the optimal catalyst binds hydrogen neither too strongly nor too weakly; Pt lies near the apex of the HER “volcano” plot owing to its outstanding intrinsic activity [33], and therefore is the most HER‐active element. HER activity is commonly benchmarked by the *j*_0_, which reflects the current under dynamic equilibrium. In practice, j_0_ is determined by fitting near‐equilibrium polarization data to the Butler–Volmer relation (or via impedance analysis under kinetic control), rather than citing “standard methods” generically. To further improve performance, heterostructure constructing is crucial [255] because it tunes the binding strength of adsorbed hydrogen (H) toward the Sabatier optimum; in alkaline media, it can also accelerate water dissociation and improve charge transfer and durability.

The Pt‑based catalysts are costly and suboptimal for alkaline HER because Pt is inefficient at cleaving the O–H bond in water. Consequently, achieving both fast water dissociation and rapid Had adsorption/conversion on Pt surfaces remains a central challenge for industrial alkaline electrolysis. Heterostructure engineering offers an effective path forward: by creating interfacial regions that (i) increase the density of metal active sites, (ii) tune H‑binding and water‑activation energetics, and (iii) lowering electrode‐scale transport losses, heterostructures can enhance Heyrovsky kinetics and simultaneously improve stability in both acidic and alkaline media [256]. Representative HER catalysts based on heterostructured materials are summarized in Table 4.

**TABLE 4:** Basic information and HER performance of heterostructured electrocatalysts in alkaline media. Tafel slopes and overpotentials were obtained from polarization curves at 10 mA cm^−^^2^. All measurements were carried out in 1 M KOH.

Electrocatalyst	Tafel slope for HER (mV dec^−1^)	Overpotential (mV)	Reference
Ru/Ni_3_Se_4_/Ni(OH)_2_/NF	33.3	—	[258]
NiO–C–MoO_2_	81.1	170	[259]
Ni_3_N/Ni	107	144	[260]
Ni_3_S_2_@NiCoN/NF	118	63	[261]
N‐MoO_2_/Cu	36	40	[262]
Co/CePO_4_	85	108	[263]
BN_3_‐NiS‐WS_2_	81	137	[264]
MoO_2_/Ni_3_S_2_/NF	85.2	74	[265]
Ru‐Ni_2_P/Fe_3_P	50.4	19.3	[266]
Co_3_Mo_3_N/Ni_3_Mo_3_N	53	36	[267]
Re_2_P/ReS_2_	66	69	[268]
Ru/W_5_N_4_	47.8	43	[269]
Ni(OH)_2_/CuMoO_4_/NF	80	70	[270]
NF/MoS_2_‐CuCo_2_S_4_	94.19	67	[271]
WS_2_/WO_3‐x_‐2	97.29	150	[272]
Co‐WS_2_	65.7	113	[273]
MoSe_2_/NiSe_2_	103	791	[274]
r‐Mn–Ni/CoP	43.3	—	[275]
Mo_2_C–W_2_C/RGO	59	87	[276]
Ni‐MoS_2_@NiS_2_@Ni_3_S_2_	—	69.4	[277]

Beyond freshwater operation, seawater electrolysis imposes distinct constraints on the cathodic HER. In addition to chloride‐related corrosion and parasitic side reactions, multivalent cations and buffering species in seawater can induce local precipitation and surface blockage under strongly alkaline cathode microenvironments, reducing active‐site accessibility and degrading mass transport at high current densities [257]. Heterostructure engineering offers a practical route to maintain fast water dissociation and optimal hydrogen adsorption while preserving electrical connectivity and interfacial robustness. For instance, a research engineered an interfacial N–TM(Co/Fe)–P electron bridge in a FeCoP/TiN nanoarray electrode, delivering 500 mA cm^−^^2^ at 152 mV and operating stably for 340 h in alkaline seawater [116]. Likewise, another research reported well lattice‐matched Co_2_Mo_3_O_8_/MoO_2_ heterointerfaces prepared via rapid Joule heating, which markedly enhance alkaline HER and enable a reported 12.4% solar‐to‐hydrogen efficiency in alkaline seawater electrolysis [257].

**FIGURE 16:**
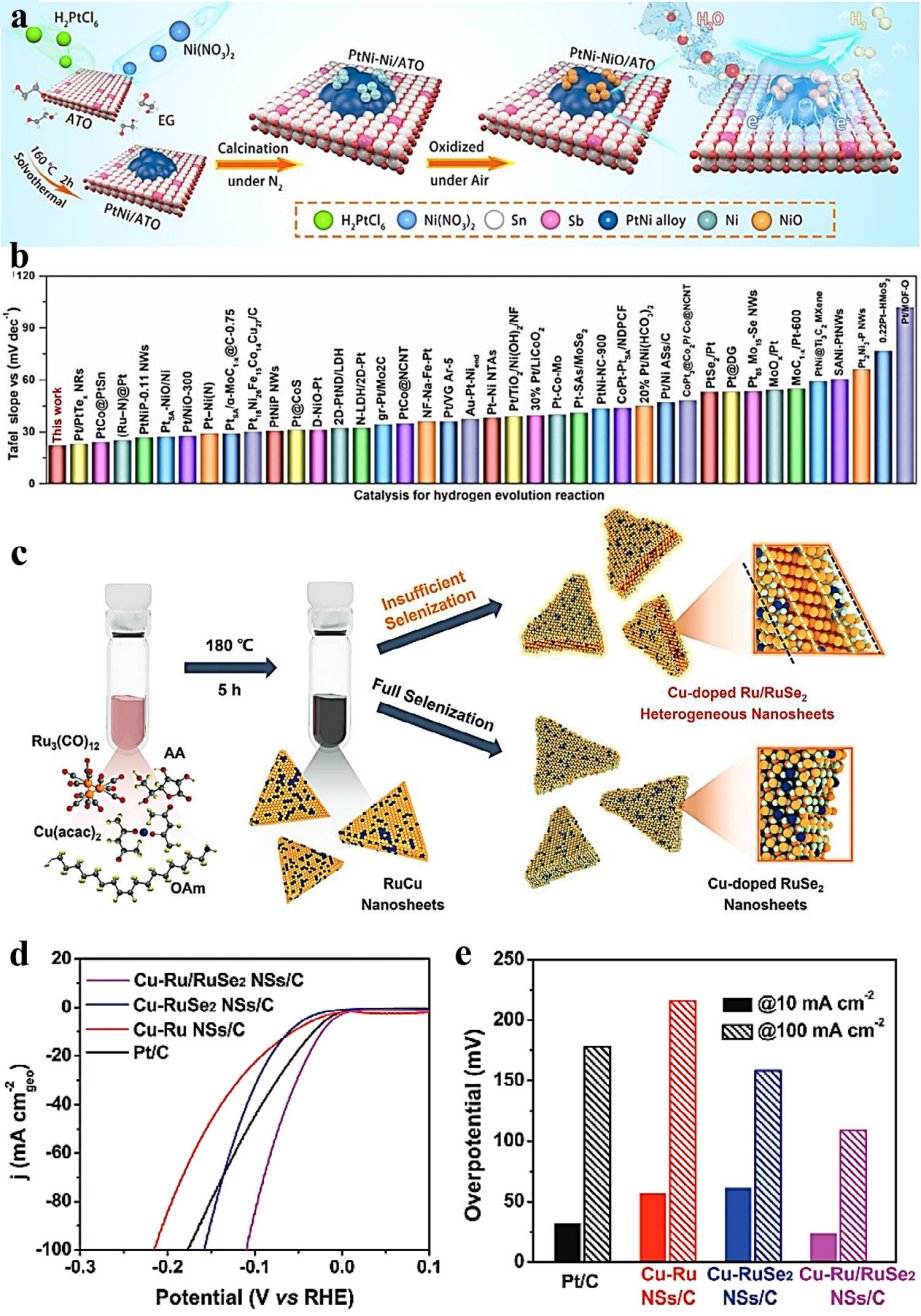
(a) Schematic depiction of the synthesis process for the ATO‐supported NiO‐PtNi heterostructures. (b) Tafel slope comparison for NiO‐PtNi/ATO with Pt‐based catalysts reported in the last three years [278] Copyright 2023, Elsevier. (c) Schematic representation of the Cu‐doped Ru/RuSe_2_ heterogeneous nanosheets and Cu‐doped RuSe_2_ nanosheets preparation. (d) HER polarization curves. (e) Overpotential at 10 and 100 mA cm^−2^ [279]. Copyright 2023, John Wiley and Sons.

**FIGURE 17:**
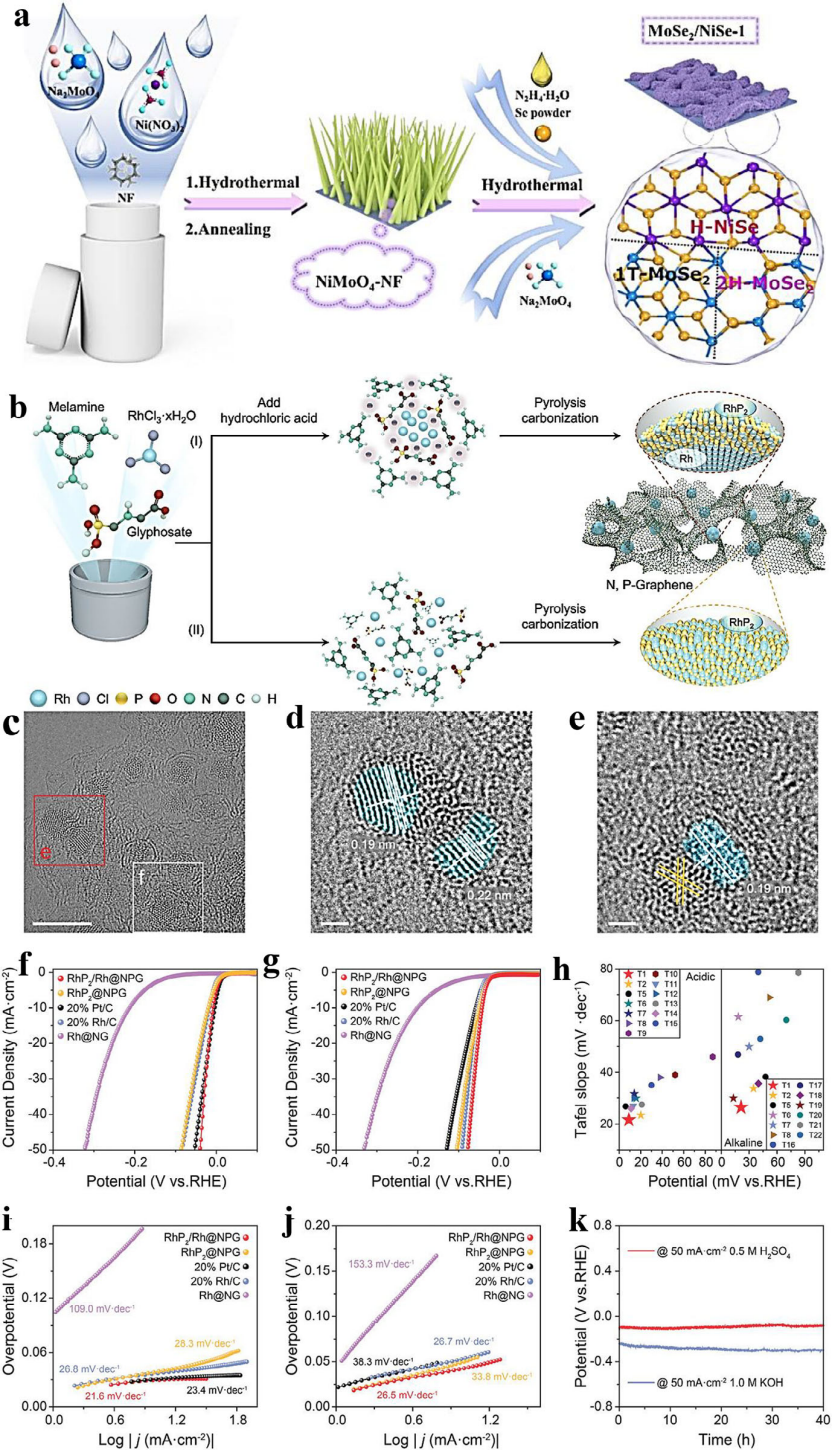
(a) Synthetic procedure for the triphase heterojunction HER electrocatalyst MoSe_2_/NiSe‐1 [280] Copyright 2022, Elsevier. (b) Schematic representation of the synthesis process for RhP_2_/Rh@NPG and RhP_2_@NPG. Abbreviations: NPG, N, P co‐doped graphene. Bright‐field high‐resolution TEM image of RhP_2_/Rh@NPG; scale bar: 5 nm. d) Enlarged view of (c); scale bar: 1 nm. e). Hydrogen evolution reaction (HER) performance. (f,g) The reversible hydrogen electrode (RHE) and internal resistance (iR)‐corrected HER polarization curves at a sweep rate of 5 mV s^−1^ in Ar‐saturated 0.5 m H_2_SO_4_ (f) and 1.0 m KOH (g) and (i,j) corresponding HER Tafel plots. (h) Comparison of overpotential (at 10 mA cm^−2^) and Tafel slope for various state‐of‐the‐art HER catalysts in acidic and alkaline media, respectively. All measurements were calibrated with iR‐compensation. Catalyst loading: 0.3 mg cm^−2^. Rotation speed: 1600 rpm. (k) Chronopotentiometric (potential–time) response for HER at a fixed current density of 50 mA cm^−2^ in acidic and alkaline electrolytes [281]. Copyright 2022, John Wiley and Sons.

An architecture featuring a NiO/PtNi heterojunction supported on antimony‑doped tin oxide (ATO) was engineered with finely controllable heterointerfaces (Figure 16a) [278]. Benchmarking the Tafel slopes against state‑of‑the‑art Pt‑based HER catalysts highlights its outstanding alkaline HER performance and underscores the advantage of constructing NiO–PtNi heterostructures on an ATO support (Figure 16b). Among Pt substitutes for HER, ruthenium selenides are especially promising. A copper‑doped Ru/RuSe_2_ hetero‑nanosheet catalyst prepared by a two‑step route (Figure 16c) optimizes the adsorption of Had and H_2_O for alkaline HER [279]. In 1.0 M KOH, the Cu‐doped Ru/RuSe_2_ heterostructured nanosheets supported on carbon (Cu–Ru/RuSe_2_ NSs/C) deliver high HER activities, requiring η of just 23 mV and 109 mV to reach 10 and 100 mA cm^−^^2^, respectively (Figure 16d,e). These values are markedly lower than those of Cu‑doped RuSe_2_ NSs/C (61, 158 mV), Cu‑doped Ru NSs/C (57, 216 mV), and even commercial Pt/C (32, 178 mV). This performance arises from precise electronic tuning accomplished by introducing a well‑defined heterointerface together with Cu doping, which jointly modulate the binding energetics of H and H_2_O at operative potentials [279]. Transition‑metal chalcogenides (TMCs) also constitute robust, earth‑abundant HER platforms. Using a hydrothermal selenization‑implantation strategy, multiphase catalysts—three‑phase MoSe_2_/NiSe‑1 and four‑phase MoSe_2_/NiSe‑2—were synthesized (Figure  17a). Both nanohybrids show enhanced activity, requiring only 30 mV and 87 mV, respectively, to reach 10 mA cm^−^^2^. DFT calculations further indicate that the MoSe_2_/NiSe‐1 junction exhibits more uniform interfacial charge redistribution than its four‐phase analogue, correlating with reduced interfacial charge‐transfer losses and faster HER kinetics [280]. This study's reported method is expected to inspire the design and preparation of more multiphase heterojunction nanocomposite materials derived from different phases (such as MX_2_, MX, and MO, where M is a metal and X is a chalcogen) for efficient electrocatalytic energy‐related reactions. In addition, Liu et al. [281] synthesized ultrafine RhP_2_/Rh nanoparticles (RhP_2_/Rh@NPG) with well‐defined semiconductor‐metal heterointerfaces. As shown in Figure 17b, the difference between the synthesis routes of RhP_2_/Rh@NPG and RhP_2_@NPG lies in the addition of HCl to the homogeneous precursor mixture, where HCl assists the self‐assembly of melamine and glyphosate via H^+^ and Cl^−^ linking. Structural characterization directly supports heterointerface formation: HRTEM images reveal a biphasic nanoscale architecture with a highly crystalline metallic Rh region (lattice fringes assignable to Rh facets) intimately coupled with a lower‐crystallinity RhP_2_ domain, indicative of RhP_2_/Rh interfacial contact within individual ultrafine particles (Figure 17c–e). The HER performance was evaluated in Ar‐saturated 0.5 M H_2_SO_4_, 1.0 M phosphate‐buffered saline, and 1.0 M KOH at a scan rate of 5 mV s^−^^1^. RhP_2_/Rh@NPG requires only 9 mV in 0.5 M H_2_SO_4_ and 21.3 mV in 1.0 M KOH to reach 10 mA cm^−^^2^, demonstrating outstanding pH‐universal activity (Figure 17f,g). Benchmarking against state‐of‐the‐art catalysts further highlights its superior kinetics, as reflected by the low overpotentials and small Tafel slopes in both acidic and alkaline media (Figure 17h–j). Moreover, the catalyst shows durable operation, exhibiting only a slight potential increase during prolonged electrolysis at a fixed current density (Figure 17k). Collectively, these results are consistent with the proposed interfacial charge‐transfer effect at the RhP_2_/Rh heterointerface, which optimizes hydrogen adsorption and simultaneously activates the dual‐site synergy of Rh and P—facilitating interfacial water reorganization or dissociation in neutral and alkaline environments and thereby accelerating HER kinetics.

In summary, across Pt‑based, Ru‑selenide, TMC, and Rh‑based systems, precise control over electronic structure and heterointerface chemistry consistently boosts HER activity and durability.

### 4.4 Nitrogen Reduction Reaction (NRR)

Ammonia (NH_3_) is not only an essential agricultural fertilizer but also a cornerstone industrial chemical. Its production is dominated by the Haber–Bosch process [282], which reacts N_2_ with H_2_ at high temperature and pressure, consuming ∼2% of global energy and emitting a substantial amount of CO_2_. Developing greener and more economical routes to NH_3_ is therefore imperative. Electrocatalytic NRR is a promising alternative that can proceed under ambient conditions. The reaction entails adsorption of N_2_ on the catalyst surface, followed by stepwise proton–electron transfers that ultimately cleave the N≡N triple bond. Realizing efficient NRR hinges on discovering catalysts that promote strong chemisorption and activation of N_2_ [35]. Transition metals are attractive because their d orbitals can interact with the π* antibonding orbitals of N_2_, lowering the effective binding energy and weakening the N≡N bond; accordingly, transition‑metal oxides, sulfides, nitrides, and carbides are widely investigated as NRR catalysts. Despite advances in NRR electrocatalysis, increasing NH_3_ yield and Faradaic efficiency remain challenging [283, 284]. The exceptional stability of N_2_ leads to weak adsorption, and the HER competes strongly for electrons and protons. To overcome these barriers, interface‑engineering strategies are being deployed to tailor adsorption energetics, reduce the activation energy for N_2_, and suppress HER, thereby improving both activity and selectivity. Guided by these principles, representative heterostructured catalysts for N_2_‑to‑NH_3_ conversion are summarized in Table 5.

**TABLE 5:** Basic information and performance of heterostructured electrocatalysts applied to NRR, where high‐purity N_2_ gas was used as the nitrogen source.

Catalyst	Electrolyte	Applied potential (V vs. RHE)	FE (%)	NH_3_ yield (µg mg^−1^_cat._ h^−1^)	Ref.
MoS_2_/C_3_N_4_	0.1 M LiClO_4_	−0.3	17.8	18.5	[285]
C@CoS@TiO_2_	0.1 M Na_2_SO_4_	−0.55	28.6	—	[286]
WS_2_/WO_2_	0.05 M H_2_SO_4_	−0.1	13.5	8.53	[287]
Mo/MoC/Mo2C	0.1 M Na_2_SO_4_	−0.3	18.9	20.4	[288]
Au_3_Cu@Cu	0.1 M Na_2_SO_4_	−0.2	21.41	33.97	[289]
BNQDs/Ti_3_C_2_T_x_	0.5 M LiClO_4_	−0.4	19.1± 1.6	52.8 ± 3.3	[290]
ZrO_2_‐BCN	0.1 M HCl	−0.65	23.1	17.8	[291]
BNQDs/C_3_N_4_	0.5 M LiClO_4_	−0.3	19.5	72.3	[292]
CoS_2_/MoS_2_	1 M K_2_SO_4_	−0.25	34.66	38.61	[293]
WSeS/WSe_2_	0.1 M Na_2_SO_4_	−0.3	40.2	15.96	[294]
Fe_2_O_3_/CuS	—	−0.2	10.6	35.67	[295]
Sv‐NiCo_2_S_4_@MnO_2_	0.1 M KOH	−0.2	20.55	57.31	[296]
ZnO/CoS	0.1 M Na_2_SO_4_	−0.2	11.7	33.03	[297]
MoO_3‐x_/MXene	0.5 M LiClO_4_	−0.4	22.3	98.5	[298]
BP/FeP_4_	0.1 M HCl	−0.2	62.9	77.6	[299]
In@Mn_3_O_4_‐5	0.1 M Na_2_SO_4_	−0.9	—	89.44	[300]

**FIGURE 18:**
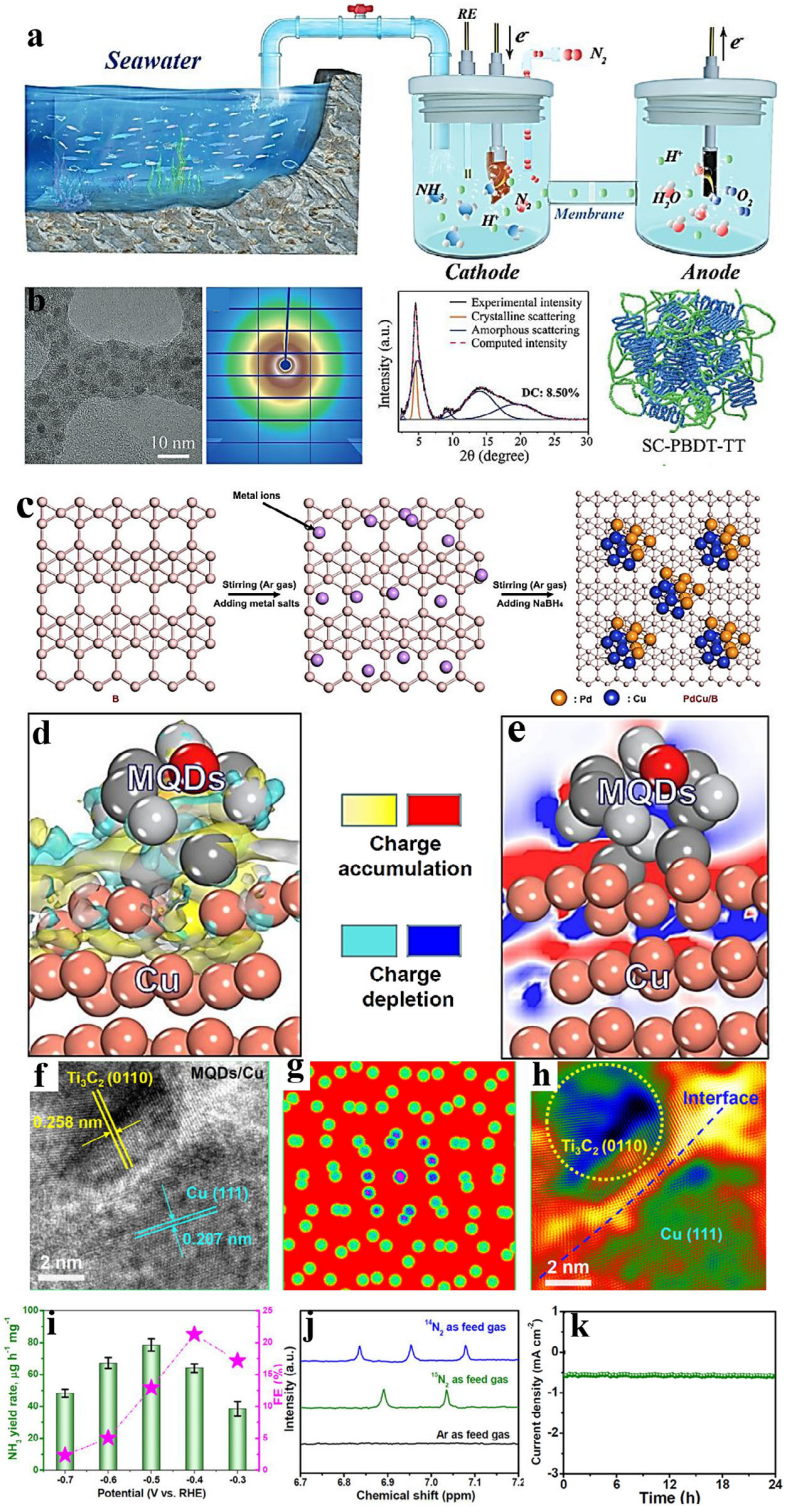
(a) Schematic depiction of neutral‐buffered seawater electrolysis. Here, RE denotes reference electrode. (b) HRTEM images and WAXD patterns acquired for SC‐PBDT‐TT, along with a 1D integrated WAXD intensity profile and corresponding peak separation analysis conducted to estimate crystallinity. Additionally, an illustration was provided to compare the microstructures of three PBDT‐TT samples with varying degrees of crystallinity, with a specific focus on SC‐PBDT‐TT [301]. Copyright 2022, John Wiley and Sons. (c) Schematic illustration of the preparation of PdCu/B [302]. Copyright 2023, John Wiley and Sons. (d) Ti_2_p spectra of MQDs/Cu and MQDs. (e) Ti_2_p spectra of MQDs/Cu and Cu. (f) HRTEM image of MQDs/Cu, and corresponding (g) masked FFT and (h) IFFT images. (i) NH_3_ yields and FEs. (j) ^1^H NMR spectra of various conditions after NRR test at −0.5 V for 2 h. (k) Long‐term chronoamperometry test [303]. Copyright 2022, John Wiley and Sons.

A recent study shows that constructing a “quasi–gas–solid” interface in donor–acceptor conjugated polymers (CPs) promotes NRR while suppressing the competing HER [301]. To conserve freshwater and avoid added salts, natural seawater was used as the electrolyte (Figure 18a). Notably, the semi‑crystalline CP catalyst SC‑PBDT‑TT delivered a Faradaic efficiency up to 60.5% and a maximum NH_3_ production rate of 16.8 µg h^−1^ mg^−1^ in neutral, buffered seawater. Structural analyses (HRTEM, 2D WAXD, and the integrated 1D WAXD profile) confirm the coexistence of crystalline and amorphous domains in SC‑PBDT‑TT (Figure 18b). To address the common activity–selectivity trade‑off, Zhang et al. [302] engineered PdCu nanoparticles with tunable Pd/Cu ratios on boron nanosheets (PdCu/B, Figure 18c). The optimal Pd1Cu1/B composition combines excellent NRR activity (overpotential 0.19 eV) with low HER activity (energy barrier 1.28 eV), illustrating how interfacial charge transfer and symmetry‑breaking sites can steer reaction pathways toward NH_3_ while disfavoring hydrogen evolution. Building on heterostructure design, another study combined Ti_3_C_2_T_x_–MXene quantum dots (MQDs) with porous copper nanosheets to yield a durable MQDs/Cu NRR catalyst [303]. The synthesis involved hydrothermal conversion of Ti_3_C_2_T_x_ to MQDs, electrostatic adsorption of Cu^2+^ onto the negatively charged MQDs, and subsequent NaBH_4_ reduction plus H_2_ annealing to form porous Cu nanosheets. HRTEM resolves dual lattice spacings attributable to Ti_3_C_2_ (MQDs) and Cu (111), and the masked FFT/IFFT analysis visualizes a well‐defined MQDs–Cu interfacial region, providing direct structural evidence for heterointerface formation (Figure 18f–h). DFT calculations—modeling a Ti_3_C_2_O_2_ MQD nanocluster on a Cu(111) slab—show electron transfer from MQDs to Cu (consistent with the lower MQD work function), with interfacial electron accumulation evidenced by charge‐density difference and the electron‐localization function (Figure 18d,e). The DOS further indicates more occupied states crossing the Fermi level at the MQDs/Cu interface, implying enhanced interfacial conductivity. Mechanistically, these electron‐rich interfaces generate Cu–Ti dimer dual sites that strongly activate N_2_ (elongated N─N bond and favorable adsorption energy) and markedly facilitate the initial hydrogenation (*N_2_ → *N_2_H), lowering the limiting barrier along the distal pathway. Experimentally, the catalyst delivers a volcano‐type potential dependence with a peak NH_3_ yield of 78.5 µg h^−^^1^ mg^−^^1^ and a maximum FE of 21.3% (Figure 18i), while ^15^N_2_ isotope‐labeled ^1^H NMR confirms NH_3_ originates from N_2_ reduction (Figure 18j) and long‐term electrolysis shows stable current retention over 24 h (Figure 18k).

In summary, these advances highlight practical levers for high‑performance electrochemical NRR: electrolyte choice (e.g., seawater), precise control of interfacial structure, and electronic tuning to enhance N_2_ activation and selectivity while suppressing HER. The insights offer clear guidance for designing robust heterojunction catalysts for sustainable NH_3_ synthesis.

### 4.5 Carbon Dioxide Electroreduction Reaction (CO_2_RR)

Carbon dioxide reduction reaction (CO_2_RR) has attracted considerable attention for its ability to convert CO_2_ into value‑added chemicals and fuels [304, 305, 306]. Although CO_2_ is thermodynamically stable, it can be activated and transformed into a spectrum of reduced products via ECR under sufficiently cathodic potentials. Mechanistically, CO_2_RR entails complex multi‑electron/proton transfer steps that involve C─O bond cleavage and C─H bond formation. Depending on the pathway, products such as carbon monoxide, methane, ethylene, formic acid, ethanol, and methanol can be obtained [307]. Catalysts are often classified by the dominant CO_2_RR product: Au and Ag tend to yield CO; Cu favors C_2_ products such as ethylene; Sn typically forms formate; whereas Pt, Fe, and Ni commonly promote hydrogen evolution. In practice, selectivity also depends on catalyst design and reaction conditions, with the adsorption energies of key intermediates playing a decisive role. A typical ECR sequence comprises (i) chemisorption of CO_2_ on the catalyst surface, (ii) activation of the C═O bond and/or formation of C─H and C─O bonds through coupled electron/proton transfer, and (iii) desorption of the product. However, high kinetic barriers associated with multi‑electron steps, together with competition from the HER, slow the overall process and complicate product control. To address these challenges, extensive efforts focus on electrocatalysts that lower activation overpotentials and increase Faradaic efficiency, among which interface modulation has emerged as a particularly effective strategy [308]. By precisely tailoring interfacial structures, catalysts can be engineered to reduce activation barriers and enhance selectivity, thereby improving the energy efficiency of CO_2_RR.

This section surveys heterostructured catalysts for CO_2_RR, with an emphasis on recent advances toward C_2_H_4_ production. We highlight how optimizing interfacial architectures promotes the key C–C coupling step, and suppresses undesired side reactions. Representative heterostructured electrocatalysts and their CO_2_‑reduction performances are summarized in Table 6.

**TABLE 6:** Basic information and properties of heterostructured materials applied to electrocatalytic CO_2_ reduction.

Catalyst	Electrolyte	Applied potential (V vs. RHE)	Target product and FE (%)	*J*_product_ (mA cm^−2^)	Reference
Cu_2_O/In(OH)_3_	0.1 M KHCO_3_	−0.95	CO(71.79)	—	[309]
Cu_1_Zn_9_−Ni	1 M KOH	−0.8	CO(81)	145	[310]
Ag–Cu NDs	0.1 M KHCO_3_	−1.1	C_2_H_4_(40)	—	[311]
Cu/CuAg	1 M KOH	−2 to −1.1	—	—	[312]
Cu−CeO_2_	1 M KOH	−0.7	C_2_H_4_O_2_(62.4)	50‐150	[313]
Cu_52_Ag_48_	1 M KOH	—	C_2_H_4_(69.2)	450	[314]
MeNiPc/G	0.5 M KHCO_3_	−0.68	CO(99)	—	[315]
MOD‐BiIn	0.5 M KHCO_3_	−1.4	HCOO^−^(97.5)	136.7	[316]
CeNCl–CeO_2_/Ni/N–C	0.5 M KHCO_3_	−0.8	CO(90)	—	[317]
Bi_2_O_3_/BiO_2_	0.5 M KHCO_3_	−1.3	HCOO^−^(98.12)	111.42	[318]
Cr_2_O_3_@Ag	0.5 M KHCO_3_	−0.8	CO(99.6)	19.0	[319]
Pd/R‐TiO_2_/TiN‐30	0.1 M KHCO_3_	−1	C_2+_(65.7)	—	[320]
Cu‐3	0.1 M KHCO_3_	−1	C_2_H_4_(68.5)	20.6	[321]
VON‐Bi‐50s‐2h	0.5 M KHCO_3_	−1	Formate(97.11)	35.6	[322]
Sm(OH)_3_/CuO	1 M KOH	−0.9	C_2_H_4_ + C_2_H_5_OH(77.2)	386	[323]
Bi–Sn	0.5 M KHCO_3_	−0.9	HCOO^−^(95.5)	11.5	[324]
Pt–Cu	0.5 M KHCO_3_	−0.7	C_3_H_6_O(66.9)	736	[325]
Cu_2_O–MgO	0.1 M KHCO_3_	−0.9	C_2_H_4_ (68.9)	27.6	[326]
CB–SnS	0.5 M KHCO_3_	−1	HCOO^−^(98.9)	51.4	[327]
Bi_2_O_2_CO_3_/CeO_2_	0.5 M KHCO_3_	−0.9	HCOO^−^(98.6)	28.6	[328]

**FIGURE 19:**
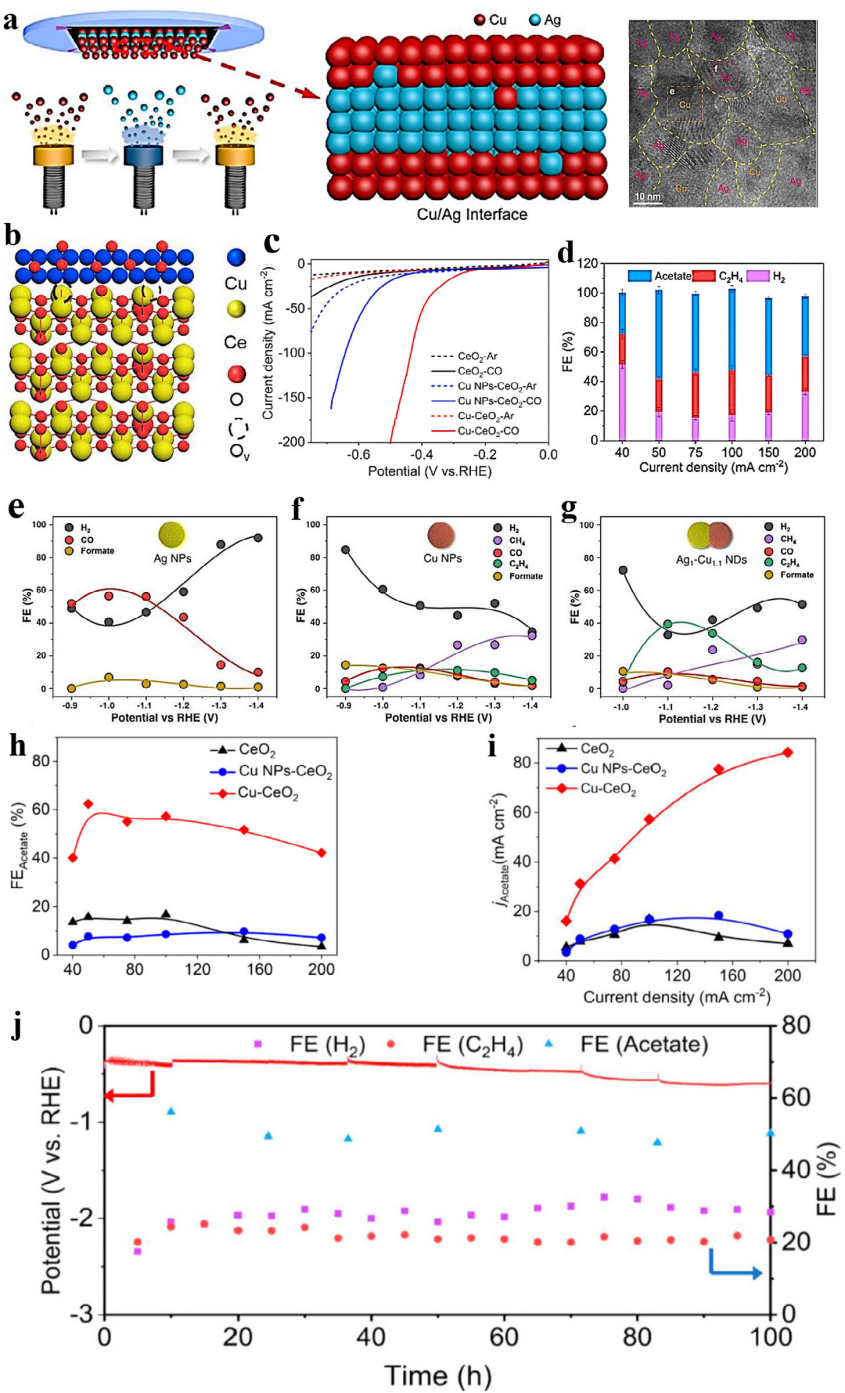
(a) Schematic depiction of the synthesis of Cu/Ag catalysts by magnetron sputtering and HRTEM images of Cu_52_Ag_48_ [314] Copyright 2024, Elsevier. (b) Schematic model of Cu−CeO_2_. (c) Linear sweep voltammetry curves of CeO_2_, CuNPs−CeO_2_, and Cu−CeO_2_ in the atmosphere of Ar and CO with 85% iR compensation. (d) Faradaic efficiencies of products on Cu−CeO_2_ [311] Copyright 2019, American Chemical Society. (e‐f) The FEs of various gaseous products, namely H_2_, CO, CH_4_, and C_2_H_4_, as well as the primary liquid product, which is formate, are depicted for: (e) silver nanoparticles (Ag NPs), (f) copper nanoparticles (Cu NPs), and (g) a composite of silver and copper nanodiscs (Ag_1_‐Cu_1.1_ NDs). (h) FEs of acetate. (i) Overlapped acetate partial current densities (*j*_p_) of CeO_2_, Cu NPs–CeO_2_, and Cu–CeO_2_ under different potentials. (j) Long‐term stability test of Cu–CeO_2_ for CORR at 50 mA·cm^−2^  [313] Copyright 2023, American Chemical Society.

**FIGURE 20:**
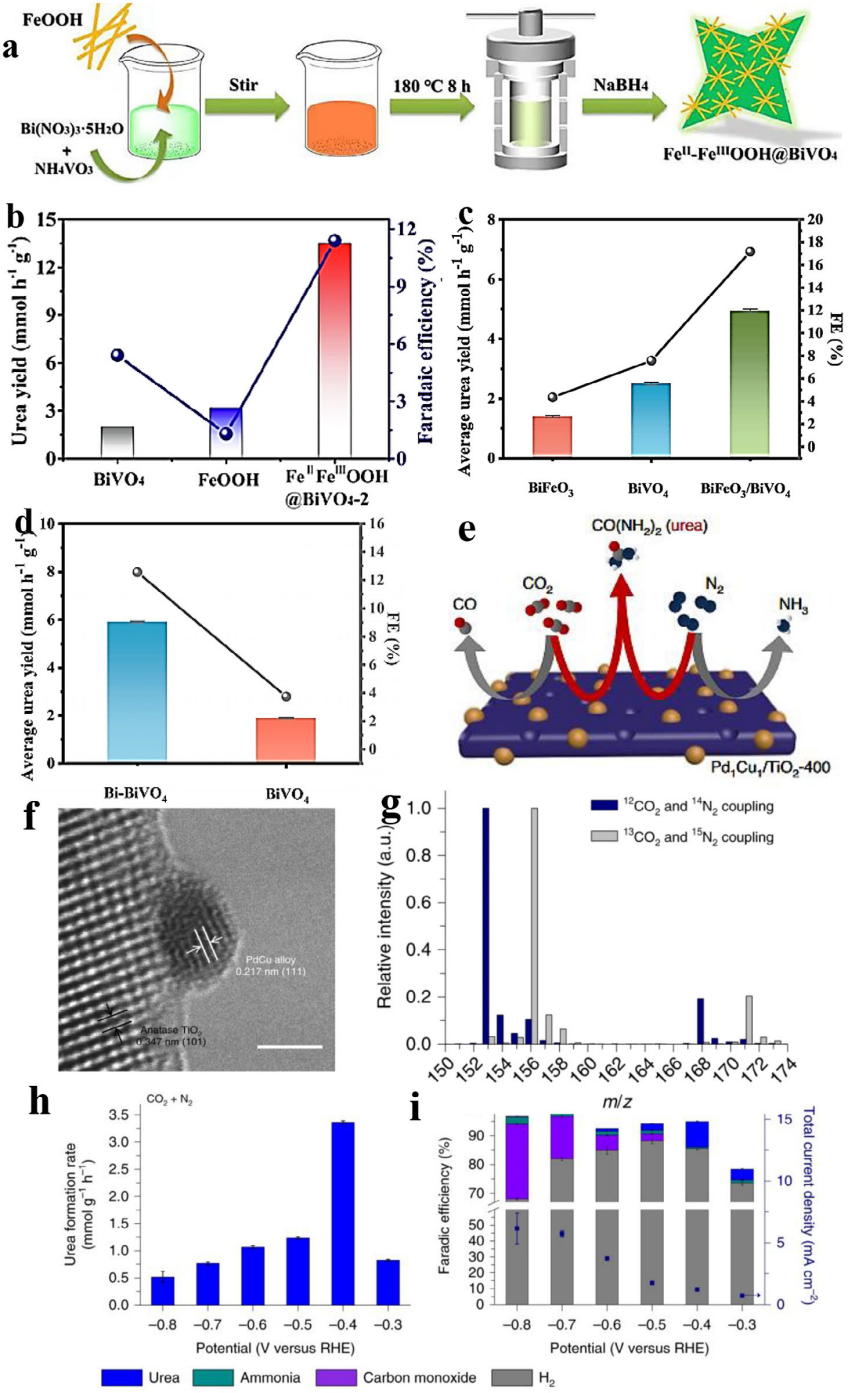
(a) Synthetic process of Fe^II^‐Fe^III^OOH@BiVO_4_ heterostructure. (b) The urea yield and FE under different applied potentials over Fe^II^Fe^III^OOH@BiVO_4_‐n [329] Copyright 2023, Elsevier. (c) Average urea yield [330] Copyright 2021, Royal Society of Chemistry. (d) Average urea yield rate and FE [331] Copyright 2021, John Wiley and Sons. (e) A schematic diagram of urea production over Pd_1_Cu_1_/TiO_2_‐400. (f) High‐resolution TEM image of Pd1Cu1/TiO_2_‐400. The scale bar is 2 nm. (g) Mass spectra of the derivation of urea with unmarked gas and isotope‐labeled gas as feeding gases. (h) Urea generation with CO_2_ and N_2_ as feeding gases. (i) The Faradic efficiencies and the total current densities for all products at various potentials [332] Copyright 2021, Springer Nature.

For the electroreduction of CO_2_ to C_2_H_4_, a Cu/Ag bimetallic catalyst (Cu_52_Ag_48_) with a strongly coupled interface has been shown to enable highly selective ethylene production at high current densities [315]. The catalyst was fabricated by sequentially sputtering thin Cu and Ag layers onto a gas‑diffusion electrode (GDE), with the Cu/Ag atomic ratio tuned via the Ag sputtering time; the atomic structure and HRTEM image are shown in Figure 19a. This work, combining experiment and theory, provides new insight into C–C coupling at the Cu/Ag interface, and the notable activity of Cu_52_Ag_48_ highlights its promise for industrial CO_2_‑to‑C_2_H_4_ conversion. In a related work, Huang et al. [311] used Ag/Cu nanocrystals to elucidate the pivotal role of the Ag/Cu interface in promoting CO_2_RR. As illustrated in Figure 20e–g, Ag–Cu nanodimers suppress CO while boosting C_2_H_4_, reaching an FE(C_2_H_4_) ≈ 40% at −1.1 V vs. RHE—a 3.4× enhancement over Cu nanoparticles and comparable to Cu nanocubes reported for (100)‐facet–rich Cu. A seeded‐growth colloidal route afforded Ag–Cu nanodimers with tunable Cu domain sizes (Ag_1_–Cu_0_._4_, Ag_1_–Cu_1_._1_, Ag_1_–Cu_3_._2_). Interfacial charge transfer—absent in physical Ag+Cu mixtures—is evidenced by a blue‐shift of the Ag SPR and XPS core‐level shifts, and together with tandem catalysis (Ag‐generated CO feeding adjacent Cu) accounts for the C_2_H_4_ enhancement [313]. As depicted in Figure 19b, interfacial Cu atoms coordinate with Ce at oxygen‐vacancy sites to form Cu–Ce(O_v_) motifs, which facilitate H_2_O adsorption/dissociation and thereby enable CO electrocoupling to acetate; performance tests (Figure 19c) show higher current densities for Cu–CeO_2_ than CuNPs–CeO_2_, and HER is strongly suppressed relative to bare CeO_2_ (Figure 19d). Consistent with the interface‐enabled suppression of competing HER and improved oxygenate selectivity, Cu–CeO_2_ delivers acetate FEs above 50% over industrially relevant current densities (50–150 mA cm^−^^2^) with a maximum FE of 62.4% at 50 mA cm^−^^2^ (Figure 19h), while simultaneously exhibiting a substantially higher acetate partial current density than CeO_2_ and CuNPs–CeO_2_ across the tested potentials (Figure 19i). Importantly, the catalyst maintains stable operation during extended electrolysis, sustaining performance over a 100 h test at 50 mA cm^−^^2^ (Figure 19j), underscoring the robustness of the Cu–Ce(O_v_) interfacial motifs under CORR conditions.

Taken together, these examples show how heterojunction catalysts can markedly improve the activity and selectivity of the CO_2_RR. Interfacial charge redistribution polarizes the C═O bonds in CO_2_, lowers activation barriers, and accelerates kinetics, thereby reducing the required overpotential. Synergistic Cu–Ag/Cu–Au heterointerfaces enhance *CO supply and interfacial charge transfer, stabilizing C─C‐coupling intermediates and favoring C_2_H_4_ formation on Cu‐based catalysts.

### 4.6 C–N Coupling Reaction

Conventional urea synthesis relies on harsh conditions, incurs high energy consumption, and raises environmental concerns. By contrast, electrochemical synthesis—driven by renewable electricity at ambient temperature and pressure—offers a sustainable alternative. In particular, coupling nitrogen sources (N_2_ or nitrates) with CO_2_ to form value‑added chemicals is promising, despite sluggish kinetics due to sizable C–N coupling barriers and low selectivity resulting from competing side reactions. To increase the selectivity, recent efforts have focused on interfacial modulation to optimize charge distribution and reaction pathways. Yin et al. synthesized an FeII‑FeIIIOOH@BiVO_4_‑n heterostructure (Figure 20a) for the co‐reduction of CO_2_ and nitrate, achieving a urea yield of 13.8 mmol g^−^^1^ h^−^^1^ with Faradaic efficiency for urea (FE(urea)) = 11.5% at −0.8 V vs. RHE, markedly higher than FeOOH or BiVO_4_ alone [329]. Building on BiVO_4_‑based junctions, Zhang et al. reported BiFeO_3_/BiVO_4_, which redistributes local charge to suppress CO poisoning and enables C–N coupling from N_2_ and CO_2_, delivering 4.94 mmol g^−^^1^ h^−^^1^ with FE(urea) = 17.18% at −0.4 V vs. RHE [330]. Similarly, Yuan et al. developed Bi–BiVO_4_ (Mott–Schottky) heterostructures, reaching 5.91 mmol g^−^^1^ h^−^^1^ with FE(urea) = 12.55% at −0.4 V vs. RHE (Figure 20b–d), again surpassing the pure components [331]. In a complementary system, a Pd_1_Cu_1_/TiO_2_‑400 electrocatalyst achieved a urea yield of 3.36 mmol g^−^^1^ h^−^^1^ with an FE of 8.92%; Figure 20e depicts the urea synthesis pathway [332]. Structural characterization confirms the formation of crystalline PdCu alloy nanoparticles, as evidenced by the resolved lattice fringes in HRTEM (Figure 20f), which underpins the intended bimetallic active motif on the OV‐rich TiO_2_ support. In flow‐cell evaluation, the catalyst exhibits a pronounced potential dependence for urea generation, delivering its highest urea formation rate at −0.4 V vs. RHE (Figure 20h) together with the corresponding Faradaic efficiencies and total current densities for all products (Figure 20i). Importantly, isotope‐labeled product identification via derivatization‐assisted GC–MS provides direct proof that both the carbon and nitrogen in the produced urea originate from the fed CO_2_ and N_2_, respectively (Figure 20g), corroborating the C–N coupling mechanism under ambient conditions. These studies indicate that heterojunction catalysts (i) activate nitrogen in N‑containing precursors via interfacial charge redistribution, thereby lowering activation barriers and accelerating kinetics; (ii) generate synergistic adsorption of key intermediates, improving selectivity for the target product; and (iii) offer enhanced structural stability and tolerance to poisoning, preserving active sites during long‑term operation—attributes essential for industrial application. Table 7 summarizes representative heterostructured catalysts for electrochemical urea synthesis.

**TABLE 7:** Basic characteristics and performance parameters of heterostructured materials for C−N coupling in electrocatalytic CO_2_ reduction.

Catalyst	Electrolyte	Applied potential (V vs. RHE)	FE (%)	Urea yield (mmol g^−1^ h^−1^)	Reference
Fe^II^‐Fe^III^OOH@BiVO_4_‐2	0.1 M KNO_3_ (NO_3_^−^)	−0.8	11.5	13.8	—
FeOOH			1.3	3.2	[329]
BiVO_4_			5.4	2.0	—
Pd_1_Cu_1_/TiO_2_‐400	0.1 M KHCO_3_ (N_2_)	−0.4	8.92	3.36	[332]
BiFeO_3_/BiVO_4_	0.1 M KHCO_3_ (N_2_)	−0.4	17.18	4.94	[330]
CeO_2_/Co_3_O_4_	0.5 M KHCO_3_ (N_2_)	—	30.05	5.81	[333]
AuPd/SnO_x_	0.075 M KHCO_3_ 0.025 M KNO_3_ (NO_3_^−^)	−0.2	21.3	2.132	[334]
Ru−Pd/WO_3_/MXene	0.5 M NaNO_3_ 0.5 M NaHCO_3_ (NO_3_^−^ + N_2_)	−0.6	23.7	—	[335]
Bi/BiVO_4_	0.1 M KHCO_3_ (N_2_)	−0.4	12.55	5.91	[331]

## 5 Summary and Outlook

**FIGURE 21:**
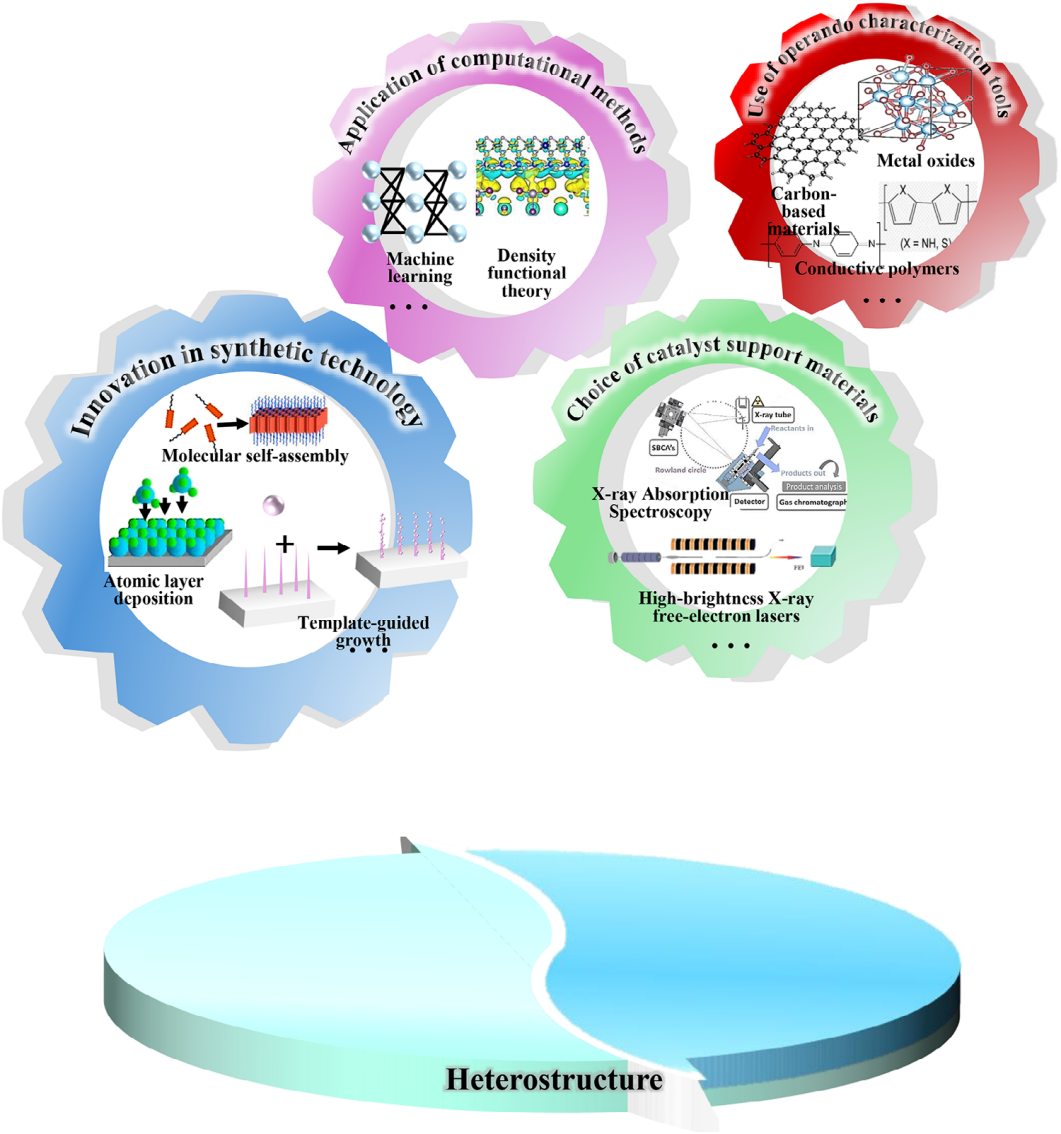
Prospects and challenges of heterostructure in electrocatalysts.

Heterostructured electrocatalysts display remarkable structural and compositional diversity, stemming from the wide design space in constituent elements and interface architectures. To present progress across reactions clearly, we compiled summary tables for each system. This review organizes progress into mechanism‐linked tables and derives a unifying framework that connects interfacial electronic structure to activity, selectivity, and durability across ORR/OER/HOR/NRR/HER/CO_2_RR. The compiled tables reveal an imbalance; recent activity is most concentrated on ORR, OER, and HER—predominantly with non‑noble metals (Fe, Co, Ni, Cu)—whereas fewer studies target HOR, NRR, and CO_2_RR. In the latter, NRR and CO_2_RR chiefly employ non‑noble catalysts, while HOR commonly relies on Ru‑ and Pt‑based materials. Mechanistically, interfacial structure governs electronic conductivity, the density and nature of active sites, and stability. We compared synthetic routes—epitaxial growth, hydro/solvothermal methods, and heterostructure coatings/arrays—and discussed how each strategy tailors interfaces to tune the performance. In essence, heterojunction enhances electrocatalysis through four levers: (i) Electronic‑structure tuning via interfacial charge redistribution; (ii) Architectural stabilization through strengthened interfacial bonding; (iii) Accelerated carrier transport along efficient interfacial pathways; and (iv) Dual‑site/interfacial adsorption that promotes key reaction intermediates. We also note that coupling with C–N, C–S, and C–P motifs broadens functionality. By offering diverse active sites and finely tunable electronic interactions, heterostructures can optimize the kinetics and thermodynamics of multi‑step transformations, increasing both yield and selectivity. Decades of research have deepened our understanding of interfacial mechanisms and guided rational catalyst design, leading to substantial performance gains. Nevertheless, component interactions remain complex and difficult to control, and structure–performance relationships in many heterostructures are still not fully resolved. Despite rapid progress, several practical bottlenecks still limit heterostructure materials from becoming fully predictive and device‐relevant. A first challenge is that interfaces are often identified qualitatively, while the density, accessibility, and chemical identity of interfacial sites are rarely quantified; as a result, performance gains can be confounded with concurrent changes in surface area, defect density, phase composition, or mass transport. Establishing quantitative descriptors—e.g., interfacial area/length per catalyst mass, junction thickness/coverage, and the fraction of electrically connected interfaces—and correlating them with measurable electrochemical metrics (ECSA, R_ct_, *j*_₀_, Tafel slope, and selectivity) will be essential for disentangling genuine interfacial effects from morphological or transport artifacts. A second, and arguably more fundamental, challenge is that the as‐synthesized interface is frequently not the working interface. Many electrocatalysts undergo potential‐ and electrolyte‐driven restructuring (surface oxidation/reduction, adsorbate‐induced segregation, hydration and (oxy)hydroxide formation, or local reconstruction at the junction), meaning that the active state can be dynamically generated, transformed, or even lost during operation [336, 337]. Consequently, mechanistic claims based solely on ex situ characterization can be incomplete or misleading. Progress therefore depends on operando/ in situ tools that can track oxidation state, coordination environment, adsorbate coverage, and interfacial electronic structure under realistic conditions (including relevant electrolyte composition, temperature, and current density), ideally combined with time‐resolved electrochemical diagnostics and model‐informed interpretation. Third, durability and translation to practical devices remain major hurdles. Heterostructures may suffer from interdiffusion, dissolution, debonding/delamination, and support corrosion, especially under high current densities, gas‐bubble stress, and start–stop potential excursions; these processes can progressively erase interfacial pathways and reduce site utilization. Beyond reporting hours of stability, future studies should diagnose failure modes through paired performance–structure tracking (e.g., retention of ECSA and R_ct_ together with post‐mortem morphology/chemistry) and should benchmark catalysts under device‐relevant configurations where local microenvironments differ sharply from idealized three‐electrode tests (e.g., gradients in pH, reactant concentration, wetting, carbonate/chloride contamination, or product crossover). This point is particularly critical for selectivity‐sensitive reactions such as CO_2_RR and NRR, where the local environment, impurities, and rigorous control experiments can dominate apparent activity and faradaic efficiency. Addressing these challenges will move heterostructure construction from an empirical “materials‐making” exercise toward a reproducible, mechanism‐grounded design discipline, thereby improving the likelihood that heterostructure catalysts deliver tangible gains in practical energy‐conversion systems. To more effectively design and optimize heterostructured electrocatalysts, we highlight several directions (Figure 21):

1. Innovation in synthetic technologies: Develop simple, controllable, scalable, and reliable methods for building well‑defined interfaces [39]. Because many materials reconstruct under operating conditions, stabilizing such interfaces throughout catalyst operation is essential for sustained performance. Specifically, interface construction should be tied to concrete levers—e.g., heterojunctions with controlled band/work‐function alignment, lattice‐matched contacting via seeded growth, and in situ exsolution/anchoring to prevent detachment and preserve charge‐transfer pathways [151, 338]. Advanced techniques—atomic layer deposition (ALD), molecular self‑assembly, template‑guided growth—enable atomic‑level interface control; additionally, external‑field modulation (electric, magnetic, stress) can dynamically tune interfaces in operando, complementing synthetic control and guiding performance toward greater efficiency, durability, and scalability.
2. Integration of computational methods: Combine ab initio molecular dynamics with periodic DFT calculations and machine learning (ML) to accelerate screening and sharpen predictions, enabling rapid identification of promising chemistries and interfaces and deeper insight into time‐dependent reaction behavior (“dynamic pathways”)—i.e., solvent‐ and field‐driven elementary steps and interfacial restructuring such as water dissociation, intermediate shuttling/diffusion, proton–electron transfer sequences, and surface/heterointerface reconstruction under operating conditions [339]. Such integrated modeling tightens feedback between theory and experiment and informs targeted synthesis.
3. Rational selection of catalyst supports: Supports must provide high conductivity and chemical/thermal stability. Carbon materials are widely used for their conductivity and surface area, yet they may degrade under oxidative or high‑temperature conditions. Developing next‑generation supports that withstand harsh operating and synthesis environments is crucial for reliable heterostructure construction and sustained activity.
4. Operando characterization and true‑site identification: While ex situ methods (HRTEM, XRD, XPS, X‐ray absorption spectroscopy) have illuminated interfacial effects, operando probes are indispensable for tracking oxidation states, charge density, and active species under realistic conditions [340, 341]. Operando XAS, Raman, and FTIR, coupled with fourth‑generation X‑ray sources, promise ultrafast, element‑specific insight into transient intermediates and interfacial electronic structures. These advances are key to pinpointing true active sites and clarifying interface evolution, thereby guiding rational interface design and synthesis.

Translating laboratory advances into practical energy‑conversion systems will require interdisciplinary collaboration spanning theory, advanced synthesis, operando diagnosis, and techno‑economic and environmental assessments. With continued innovation in heterostructure constructing—supported by scalable fabrication, predictive computation, and incisive operando tools—heterostructured electrocatalysts are poised to accelerate the sustainable conversion and storage of clean energy and to contribute meaningfully to decarbonization.

## Conflicts of Interest

The authors declare no conflicts of interest.

## Data Availability

The authors have nothing to report.
